# EIF4A3‐Induced Circular RNA circSnd1 Promotes Muscle Atrophy and Muscle Ageing by Stabilizing EEF1A1

**DOI:** 10.1002/jcsm.70210

**Published:** 2026-01-29

**Authors:** Jin Li, Bing Jin, Yuwei Yan, Yuying Chen, Xiaohang Yin, Xinyi Ren, Qian Li, Jingying Chen, Siqi Wang, Tingting Yang, Yanan Zhang, Qiumeng Nie, Dongchao Lu, Ming Wu, Yan Yu, Lei Chen, Tarun Keswani, Guoping Li, Dragos Cretoiu, T. Scott Bowen, Junjie Xiao, Yongjun Zheng

**Affiliations:** ^1^ Cardiac Regeneration and Ageing Lab, Institute of Geriatrics (Shanghai University) Affiliated Nantong Hospital of Shanghai University (The Sixth People's Hospital of Nantong) and School of Life Sciences, Shanghai University Nantong China; ^2^ Institute of Cardiovascular Sciences, Shanghai Engineering Research Center of Organ Repair, Joint International Research Laboratory of Biomaterials and Biotechnology in Organ Repair (Ministry of Education), School of Life Sciences Shanghai University Shanghai China; ^3^ School of Integrative Medicine Shanghai University of Traditional Chinese Medicine Shanghai China; ^4^ Department of Orthopedics Shanghai Gongli Hospital Shanghai China; ^5^ Department of Spine Surgery, Tongji Hospital, School of Medicine Tongji University Shanghai China; ^6^ Center for Immunological and Inflammatory Diseases, Department of Medicine Massachusetts General Hospital and Harvard Medical School Boston Massachusetts USA; ^7^ Cardiovascular Division of the Massachusetts General Hospital and Harvard Medical School Boston Massachusetts USA; ^8^ Department of Medical Genetics Carol Davila University of Medicine and Pharmacy Bucharest Romania; ^9^ Materno‐Fetal Assistance Excellence Unit Alessandrescu‐Rusescu National Institute for Mother and Child Health Bucharest Romania; ^10^ School of Biomedical Sciences, Faculty of Biological Sciences University of Leeds Leeds UK; ^11^ Division of Pain Management Huadong Hospital Affiliated to Fudan University Shanghai China

**Keywords:** circSnd1, EEF1A1 ubiquitination, EIF4A3, FAT10, muscle ageing, muscle atrophy

## Abstract

**Background:**

Muscle atrophy is a common complication of ageing, and many chronic conditions, lacks defined therapeutic interventions. It is still mostly unknown how circular RNAs contribute to muscle atrophy.

**Methods:**

circRNA sequencing and quantitative real‐time PCR were performed to detect the changed circRNAs in muscle atrophy models and aged muscle. Then the gain‐of‐function and loss‐of‐function experiments were used to investigate the function of circSnd1 in muscle atrophy and muscle ageing. Furthermore, we used RIP‐MS and RIP assay to determine the downstream and upstream mechanism of circSnd1 in muscle atrophy.

**Results:**

Here, we characterized the function and mechanism of highly species‐conserved circRNA derived from staphylococcal nuclease and Tudor domain containing 1 gene (named circSnd1) in muscle atrophy. CircSnd1 is upregulated in many types of muscle atrophy models in both in vivo and in vitro (all *p* < 0.01). Meanwhile, circSnd1 is also higher expressed in aged muscle in humans (+2.2‐fold, *n* = 5, *p* < 0.05), mice (+43.96%, *n* = 6, *p* < 0.05) and myotubes (+42.21%, *n* = 6, *p* < 0.05). Functional analyses show that circSnd1 promotes muscle atrophy and muscle ageing at the cellular level and mouse level while repressing it ameliorates multiple types of muscle atrophy (all *p* < 0.05). Mechanistically, the RNA binding protein eukaryotic translation initiation factor 4A3 (EIF4A3) can bind to the intron flanking sequence of circSnd1 to induce circSnd1 cyclization and increase circSnd1 expression in muscle atrophy. In addition, circSnd1 promotes the binding between human HLA‐F adjacent transcript 10 (FAT10) and eukaryotic translation elongation factor 1 alpha 1 (EEF1A1). FAT10 competes with ubiquitin for binding with EEF1A1, which decreases the ubiquitination of EEF1A1 and stabilizes the protein level of EEF1A1 in muscle cells to promote atrophy.

**Conclusions:**

We have identified circSnd1 as a novel circRNA that promotes muscle atrophy and highlighted its potential as a novel therapeutic target.

## 1 Introduction

Skeletal muscle atrophy leads to the loss of muscle strength as well as the reduction of muscle mass, which is majorly caused by excessive protein degradation [1, 2]. Ageing (sarcopenia), inactivity (immobilization), denervation and chronic disorders (heart failure, renal failure, chronic obstructive pulmonary disease and cancer cachexia etc.) are all linked to muscle loss [3]. The major issues of patients suffering from muscle atrophy are the diminished quality of life, the elevated (re‐)hospitalization ratio with high frequency of complications and the increased mortality [4, 5]. However, no effective pharmacological method can cure muscle atrophy currently. Therefore, to find new treatment, a deeper comprehension of the processes behind muscle atrophy is desperately needed.

Circular RNAs (circRNAs) are a unique class of covalently closed RNAs that lack tails. Unlike traditional linear RNA, most circRNAs are produced from the exons of precursor mRNAs and form a circular structure [6]. CircRNAs are very stable and frequently show expression patterns unique to various cell types and tissues [7]. CircRNAs are involved in a variety of biological processes. CircRNAs can modulate transcription by interfering with splicing, sponging or scaffolding macromolecules. Furthermore, circRNAs also code for new polypeptides or proteins [8]. CircRNAs play a crucial role in various diseases, including cancer, cardiovascular diseases and neurological disorders [9]. In addition, many circRNAs are involved in myogenesis and muscle development, such as circZNF609, circFgfr2, circIGF1R, and circGPD2 [S1–S4]. Emerging evidence indicates some circRNA dysregulation in disease‐induced muscle atrophy, such as circTmeff1, circDdb1, circANAPC7 and circBBS9 [10, 11, 12, 13].

Aside from acting as miRNA sponges, circRNAs have the potential to function as protein sponges, engaging with RNA binding proteins (RBPs) and controlling gene expression [14]. Additionally, circRNAs have been identified as a newly recognized class of regulators that have been reported to function as protein decoys, scaffolds, and recruiters [15]. For example, mannan binding lectin (MBL) protein promotes circMbl biogenesis by binding to circMbl flanking introns and then circMbl can sponge MBL proteins, thus regulating the function between the circMbl and MBL protein by a feedback loop regulation module [S5]. Another circRNA, SCAR, localized in mitochondria, can bind ATP synthase F1 subunit beta (ATP5B), thus blocking the mitochondrial permeability transition pore (mPTP) opening and maintaining mROS homeostasis [S6]. CircFoxo3 forms a ternary complex by binding with p21 and CDK2, leading to the arrest of the CDK2 protein. Thus, in turn, inhibits cell cycle entry [S7]. By stimulating Ybx1 degradation and reinforcing its association with the E3 ubiquitin ligase Nedd4l, CircNfix suppresses the proliferation of cardiomyocytes [S8]. Some RBPs regulate circRNA biogenesis by interacting with circRNA flanking introns. Heterogeneous nuclear ribonucleoprotein L (HRNPL), fused in sarcoma (FUS) and RBP quaking I (QKI) can promote the formation of circRNAs, whereas adenosine deaminase 1 (ADAR1) disturbs the formation of complementary structures of flanking sequences, thus repressing the circRNA formation [S9–S12]. Therefore, it is necessary to further explore the regulation between RBPs and circRNAs for the certain conditions including muscle atrophy.

In this study, we discovered a circRNA originating from the exons of the coding gene staphylococcal nuclease and Tudor domain containing 1 (SND1), named circSnd1 (mmu_circ_0013252), which is the novel regulator of muscle atrophy. We discovered that muscle atrophy is linked to the upregulation of circSnd1. In vivo and in vitro phenotypic research demonstrated that mimicking the upregulation of circSnd1 in skeletal muscle could lead to muscle atrophy and muscle ageing while repressing circSnd1 alleviated muscle atrophy. Mechanistically, circSnd1 promoted muscle atrophy by binding with eukaryotic translation elongation factor 1 alpha 1 (EEF1A1). Moreover, the circSnd1 expression was upregulated by RBP eukaryotic translation initiation factor 4A3 (EIF4A3) in muscle atrophy. Based on our findings, it can be inferred that circSnd1 may represent a promising therapeutic target for muscle atrophy.

## 2 Materials and Methods

### 2.1 Animal Models

We obtained 8‐week‐old male C57BL/6 mice from Charles River (Beijing, China) and housed them in the SPF Laboratory Animal facility at Shanghai University. All animal testing procedures are in accordance with the NIH Guidelines for the Use and Care of laboratory Animals for Biomedical Research (no. 85‐23, revised in 1996), and the experimental protocol was approved by the Ethics Committee of Shanghai University (approval number: ecshu2020‐100). Muscle atrophy models were developed in accordance with our prior research [16, S13–S18]. In short, sciatic nerves in the right hind limb of mice were severed for denervation‐induced muscle atrophy; sham animals had the same procedure but without sciatic nerve amputation. In order to offer a 90° fixation of the mouse hindlimb for immobilization‐induced muscle atrophy, a 0.4 × 8 mm size screw was inserted into the tibial shaft through the calcaneus and talus; hind limbs of non‐immobilized mice were selected as sham controls. Ang II (Angiotensin II human Acetate, Selleck, TX, USA) was implanted into mice for 1 week at a rate of 1.46 mg/kg/day using an osmotic minipump to treat Angiotensin II‐induced muscle atrophy (ALZET, Cupertino, CA, USA). The mice in the control group were given an implant of a PBS osmotic pump. The mice were sacrificed, and the gastrocnemius of the right hind limb was collected for further analysis after 1 week of the muscle atrophy model construction.

### 2.2 Human Samples

Skeletal muscle tissue near the knee joint was collected with the use of arthroscopy during the operation in the Shanghai Gongli Hospital. The participants were split into groups based on their age, with one group being young and the other ageing. All procedures conformed to the 1964 Helsinki Declaration and its later amendments or comparable ethical standards and were approved by the Ethics Committee of Shanghai Gongli Hospital (GLYYls2024‐016). Each subject provided written informed consent. The information of muscle sample donors was listed in our previous work [12].

Other methods were found online in the Supporting Information.

### 2.3 Statistical Analysis

Analytical statistics results were presented using GraphPad Prism 8.0 as mean ± SD The comparison between the two groups was conducted by independent sample *t*‐test. Two‐factor ANOVA and Tukey test were used for multiple groups of samples, or one‐way ANOVA and Dunnett's T3 or Bonferroni tests were used for homogeneity of variance. Results with *p* < 0.05 were deemed to be significant.

## 3 Results

### 3.1 Elevated Expression of circSnd1 in Muscle Atrophy

**FIGURE 1:**
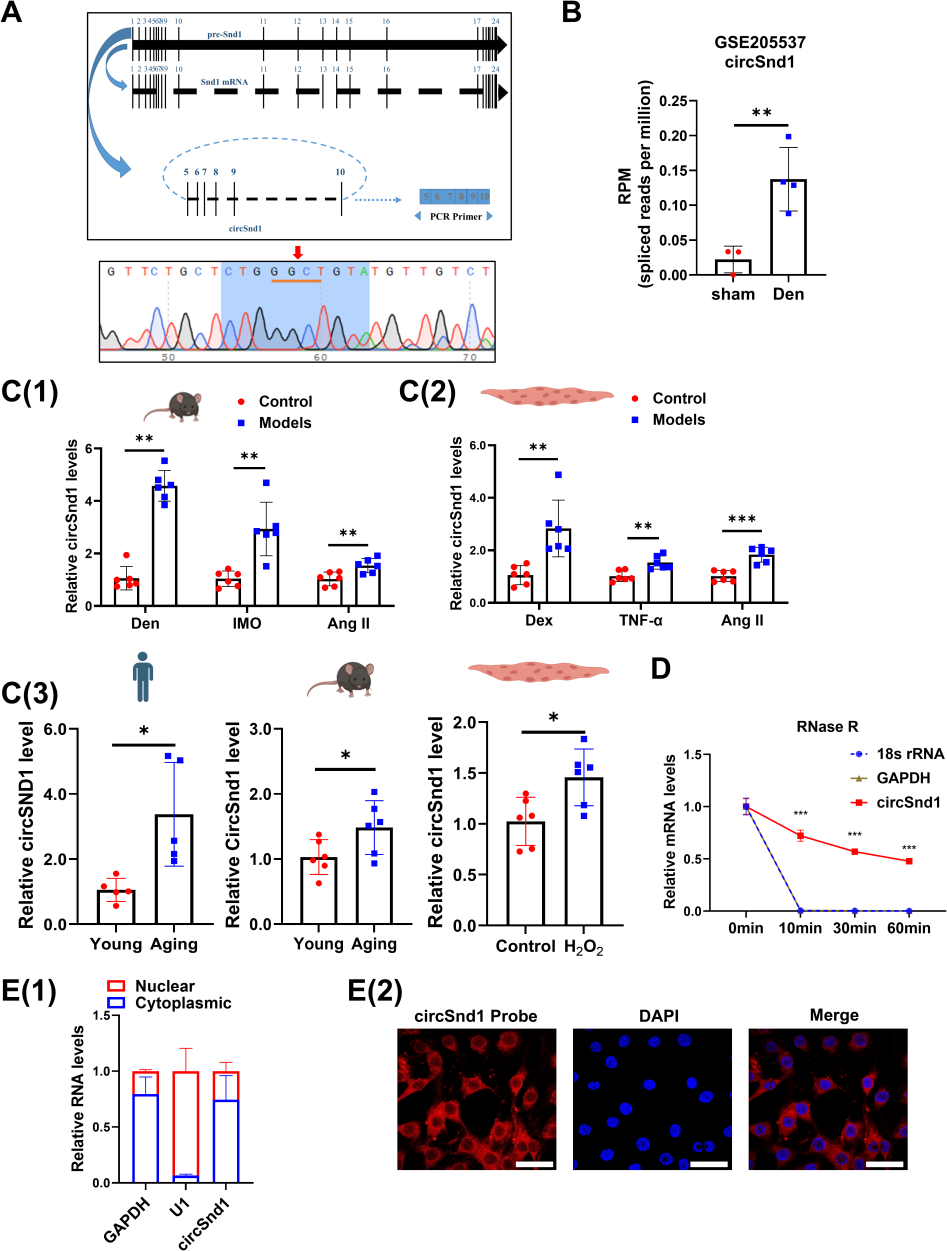
Circular RNA circSnd1 is upregulated in muscle atrophy and aged muscle. (A) The circSnd1 structure. Sanger sequencing analysis the reverse cleavage site of circSnd1 in mice. (B) GSE205537 dataset was analysed by GEO2R, and circSnd1 expression values were determined in denervation (Den)‐induced muscle atrophy in mice (*n* = 3, 4). (C) RNA expression levels of circSnd1 in muscle atrophy models. (1) RNA expression levels of circSnd1 in gastrocnemius muscle samples from muscle atrophy mice that suffer from denervation (Den), immobilization (Imo) and angiotensin II (AngII) treatment (*n* = 6 per group). (2) RNA expression levels of circSnd1 in C2C12 myotubes treated with Dex, TNF‐α and AngII (*n* = 6 per group). (3) RNA levels of circSnd1 in muscle samples from aged human (*n* = 5 per group), aged mice (*n* = 6 per group) and H_2_O_2_‐induced myotube atrophy model (*n* = 6 per group). (D) RNA levels of circSnd1, 18s rRNA and linear mRNA GAPDH when treated with RNase R for 0, 10, 30 and 60 min (*n* = 4 per group). (E) (1) The nuclear and cytoplasmic circSnd1 content in C2C12 myotubes (*n* = 6 per group). (2) The nuclear and cytoplasmic circSnd1 content measured by FISH experiments (scale bar: 10 μm); red, circSnd1; blue, DAPI. An unpaired, two‐tailed Student's *t*‐test was used (B–E). *p < 0.05; **p < 0.01; ***p < 0.001. Data are represented as mean ± SD.

CircSnd1 (circBase ID: mmu_circ_0013252; spliced sequence length: 724; position: chr6:28476052‐28495598) was shown to be a circRNA formed by cyclization of exons 5–10 of Snd1 in mice (Figure 1A). CircSnd1 was highly conserved between human (circBase ID: hsa_circ_0082113; spliced sequence length: 724; position: chr7:127341216‐127361454; strand: +) and mice (Figure S1). In addition, circSnd1 is widely expressed in different organs, including the muscle system (skeletal muscle, heart muscle and smooth muscle) (Figure S2A,B). CircSnd1 was upregulated in vivo during atrophy‐induced denervation according to our previous high‐throughput RNA‐sequencing (RNA‐seq) data in the Gene Expression Omnibus (Accession Number: GSE205537, https://www.ncbi.nlm.nih.gov/geo/query/acc.cgi?acc=GSE205537) (Figure 1B) [13]. To investigate whether dysregulated circSnd1 might play a functional role during muscle atrophy, we first examined the circSnd1 level in various muscle atrophy models in vitro and in vivo. Divergent primers, spanning the back‐splicing site, were designed and used to measure circSnd1 expression in multiple types of muscle atrophy both in vivo (denervation, angiotensin II [Ang II] and immobilization [Imo]‐induced muscle atrophy) and in vitro (dexamethasone [Dex], tumour necrosis factor alpha [TNF‐α] and Ang II‐induced muscle atrophy) by qRT‐PCR. CircSnd1 expression was significantly increased in these muscle atrophy models, whereas Snd1 linear mRNA expression level was not changed (Figure 1C(1) and (2) and Figure S2C–H). Besides that, to explore whether the upregulation of circSnd1 in muscle atrophy was specific in muscle fibre type, we analysed circSnd1 expression level in other type muscles (Type I: soleus; Type II: gastrocnemius; combination of Type II and Type I: tibialis anterior) after muscle atrophy. We found that circSnd1 was elevated in all these muscles in muscle atrophy (Figure S2I). Especially, CircSnd1 expression was upregulated in aged human muscle, mice muscle and myotube (Figure 1C(3)). After checking the expression of circSnd1 in these muscle atrophy models, the Sanger sequencing for the back‐splicing site and RNase R treatment were introduced to determine the circular structure of circSnd1. CircSnd1 showed the junction site and resistance to RNase R treatment (Figure 1A,D). In addition, the circSnd1 transcript was observed in the cytoplasm of C2C12 myoblast by using subcellular fractionation assays and fluorescence in situ hybridization (FISH) (Figure 1E).

### 3.2 CircSnd1 Induces Muscle Atrophy and Muscle Ageing In Vitro and In Vivo

**FIGURE 2:**
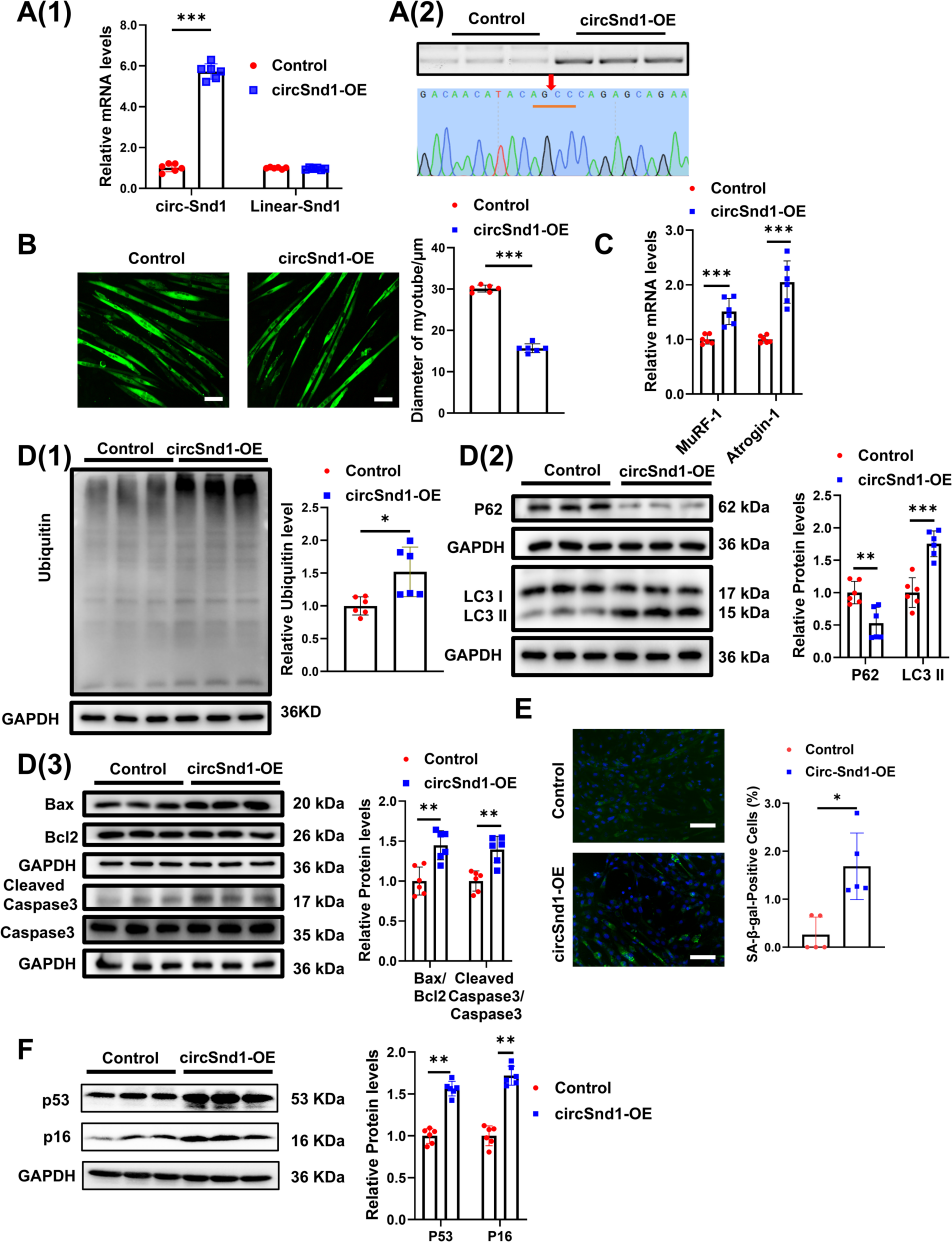
CircSnd1 promotes muscle atrophy and muscle ageing in vitro. (A) (1) RNA levels of circSnd1 and linear Snd1 expression in circSnd1‐OE C2C12 myotubes compared to control group (*n* = 6 per group). (2) Analysing the cirSnd1 overexpression by performing gel electrophoresis and sequencing of the back‐splicing junction. (B) Performing immunofluorescent staining (*n* = 6 per group; scale bar: 50 μm) and measuring the diameter in circSnd1‐OE C2C12 myotubes in comparison to the control group (*n* = 6 per group). (C) mRNA levels of MuRF‐1 and Atrogin‐1 in circSnd1‐OE C2C12 myotubes compared to control group (*n* = 6 per group). (D) Protein level of ubiquitin (1), P62 and LC3 II (2), Bax, Bcl2 and Caspase3 (3) in circSnd1‐OE C2C12 myotubes compared to control group (*n* = 6 per group). (E) SA‐β‐gal staining in circSnd1‐OE C2C12 myotubes (*n* = 5 per group; scale bars, 50 μm). (F) Protein levels of P53 and P16 in circSnd1‐OE C2C12 myotubes compared to control group (*n* = 6 per group). An unpaired, two‐tailed Student's *t*‐test was used (A–F). *p < 0.05; **p < 0.01; ***p < 0.001. Data are represented as mean ± SD.

After confirming the elevated expression of circSnd1 in muscle atrophy, the effects of overexpression of circSnd1 were explored in C2C12 myotube cells. CircSnd1 overexpression plasmid markedly increased the expression of circSnd1, without affecting Snd1 linear mRNA expression in the C2C12 myotube cells (Figure 2A). The smaller size of myotube induced by circSnd1 overexpressing plasmid compared to control plasmid was observed by immunofluorescence (Figure 2B). Furthermore, the atrogenes Atrogin‐1 and MuRF‐1 were upregulated in circSnd1 overexpressing plasmid myotube (Figure 2C). These results indicate that high circSnd1 expression triggers muscle atrophy in vitro.

Then, the degradation pathways in circSnd1 overexpressed C2C12 myotubes were evaluated by western blot and RT‐qPCR. Ubiquitin‐proteasome system (UPS) was enhanced in the setting of circSnd1 overexpression (Figure 2D(1) and Figure S3A). Moreover, examination of autophagy‐associated proteins and gene expression revealed that circSnd1 overexpressed C2C12 myotubes exhibited activation of the autophagy‐lysosome pathway (ALP) (Figure 2D(2) and Figure S3B). In addition, apoptosis was enhanced when C2C12 myotubes were transfected with circSnd1 overexpression plasmid, which was confirmed by western blot (Figure 2D(3)). In contrast, the protein synthesis related AKT‐FOXO3A‐mTOR signalling pathway was inactivated when circSnd1 was overexpressed in C2C12 myotubes as evidenced by the reduction of the phosphorylation level of AKT (S473), FOXO3A (S253), mTOR, P70S6K and EIF‐4EBP1 (Figure S3C). Furthermore, the high level of circSnd1 was linked to a lower mitochondrial DNA (mtDNA) copy number in myotubes (Figure S3D). Taken together, circSnd1 appears to increase protein degradation to promote muscle atrophy in vitro. To better fit the clinical situation, we examined the function of circSND1 in human skeletal muscle myoblasts induced to myotubes. The results also showed that circSND1 overexpression promoted muscle atrophy in myotubes differentiated from human skeletal muscle myoblasts (Figure S4).

In addition, an increase in senescence‐associated beta‐galactosidase (SA‐β‐gal) activity was detected in circSnd1‐overexpressed myotube (Figure 2E). Meanwhile, the upregulated expression of P53 and P16 was stimulated under circSnd1 overexpression (Figure 2F). These data indicated accelerated senescence of myotubes with circSnd1 overexpression.

**FIGURE 3:**
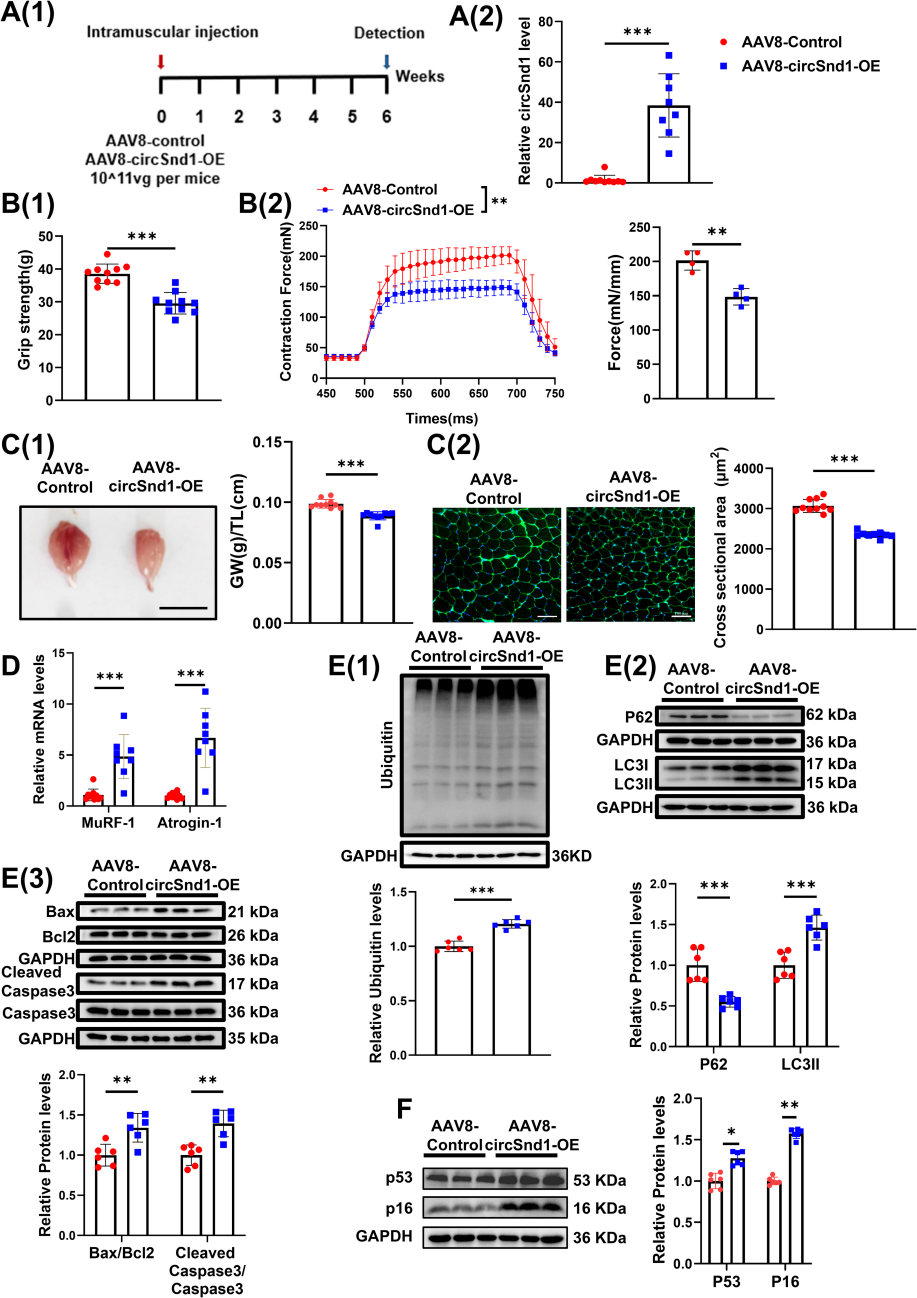
CircSnd1 promotes muscle atrophy and muscle ageing in vivo. (A) (1) Schematic diagram of experimental design. (2) RNA level of circSnd1 expression in mouse gastrocnemius muscle treated with circSnd1‐OE (circSnd1 overexpression) AAV8 (AAV8‐circSnd1‐OE) compared to the control AAV8 (*n* = 8–10 per group). (B) Muscle function tests. (1) The grip strength of right hind limb of mouse treated with AAV8‐circSnd1‐OE compared to the control AAV8 (*n* = 10 per group). (2) Muscle tetanic contraction of mouse treated with AAV8‐circSnd1‐OE compared to the control AAV8 (*n* = 4 per group). (C) The size of muscle and myofibre of mice. (1) Gastrocnemius muscle morphology and weight (GW)/tibia length (TL) ratio in mouse gastrocnemius muscle treated with AAV8‐circSnd1‐OE compared to the control AAV8 (*n* = 10 per group; scale bar: 1 cm). (2) WGA staining for myofibre of mouse gastrocnemius muscle treated with AAV8‐circSnd1‐OE compared to the control AAV8 (*n* = 8 per group; scale bar: 100 μm). (D) mRNA levels of MuRF‐1 and Atrogin‐1 expression in mouse gastrocnemius muscle treated with AAV8‐circSnd1‐OE compared to the control AAV8 (*n* = 8 per group). (E) Protein level of ubiquitin (1), P62 and LC3 II (2), Bax, Bcl2 and Caspase3 (3) in mouse gastrocnemius muscle treated with AAV8‐circSnd1‐OE compared to the control AAV8 (*n* = 6 per group). (F) Protein level of P53 and P16 in mouse gastrocnemius muscle treated with AAV8‐circSnd1‐OE compared to the control AAV8 (*n* = 6 per group). An unpaired, two‐tailed Student's *t*‐test was used (A–F). *p < 0.05; **p < 0.01; ***p < 0.001. Data are represented as mean ± SD.

To test the pro‐atrophy function of circSnd1 in vivo, we constructed AAV8‐circSnd1 and AAV8‐control viral particles and delivered them into adult male mice by intramuscular injection followed by phenotyping 6 weeks later (Figure 3A(1)). AAV8‐circSnd1‐mediated treatment led to a significant upregulation of circSnd1 expression in gastrocnemius muscle (Figure 3A(2)). In vivo muscle function showed a significant reduction of grip strength in AAV8‐circSnd1‐treated mice (Figure 3B(1)). After circSnd1 overexpression, the tetanic forces of isolated EDL muscles were lower than those of the AAV8 control (Figure 3B(2)). Consistent with this result, we further found that the gastrocnemius muscle weight and myofibre cross‐sectional area (CSA) were also smaller in mice treated with AAV8‐circSnd1 (Figure 3C). These data suggested that overexpression of circSnd1 reduced both muscle function and muscle mass. As expected, the atrogenes Atrogin‐1 and MuRF‐1 were upregulated in muscle from circSnd1‐treated mice (Figure 3D), alongside activation of the UPS, ALP and apoptosis (Figure 3E and Figure S5A,B). Furthermore, the AKT‐FOXO3A‐mTOR signalling pathway was also inactivated in circSnd1‐overexpressed mice (Figure S5C). In addition, circSnd1 overexpression did not alter the myofibre composition in muscle from circSnd1‐treated mice (Figure S5D). Meanwhile, upregulated expression of P53 and P16 was observed in muscle from circSnd1‐treated mice (Figure 3F), which indicated accelerated ageing of muscle by circSnd1 overexpression. Collectively, these data indicate that circSnd1 can induce muscle atrophy in vivo.

### 3.3 Loss of circSnd1 Attenuates Muscle Atrophy In Vitro and In Vivo

**FIGURE 4:**
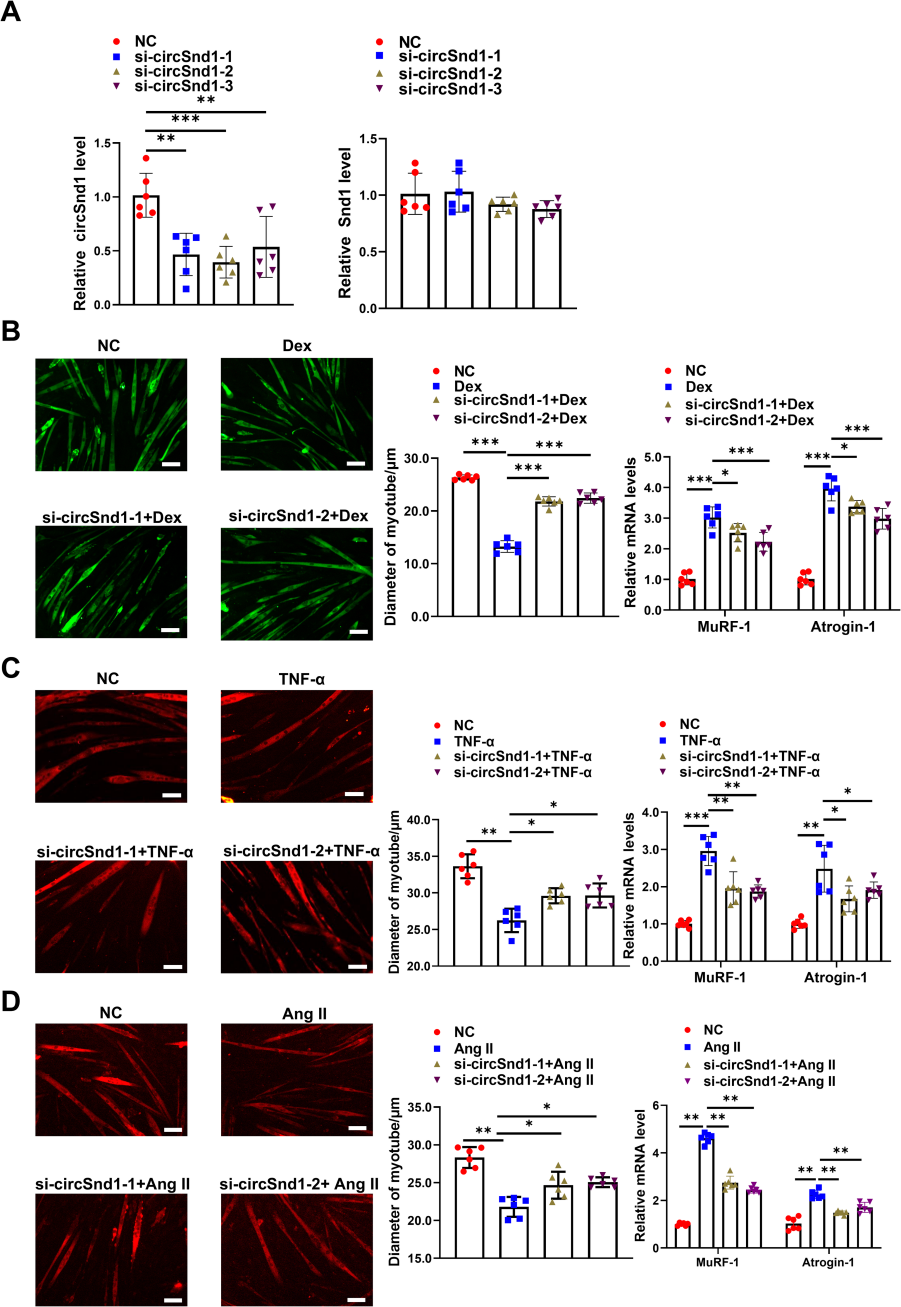
Inhibition of circSnd1 expression prevents muscle atrophy in vitro. (A) RNA levels of circSnd1 and Snd1 expression in C2C12 myotubes transfected with si‐circSnd1‐1, si‐circSnd1‐2 and si‐circSnd1‐3 (*n* = 6 per group). (B) Left: Performing immunofluorescent staining and measuring the diameter of C2C12 myotubes transfected with si‐circSnd1‐1 and si‐circSnd1‐2 in dexamethasone (Dex)‐induced muscle atrophy model (*n* = 6 per group; scale bar: 50 μm). Right: MuRF‐1 and Atrogin‐1 (*n* = 6 per group) mRNA expressions in C2C12 myotubes transfected with si‐circSnd1‐1 and si‐circSnd1‐2 in dexamethasone (Dex)‐induced muscle atrophy model. (C) Left: Performing immunofluorescent staining and measuring the diameter of C2C12 myotubes transfected with si‐circSnd1‐1 and si‐circSnd1‐2 in tumour necrosis factor alpha (TNF‐α)–induced muscle atrophy model (*n* = 6 per group; scale bar: 50 μm). Right: MuRF‐1 and Atrogin‐1 (*n* = 6 per group) mRNA expressions in C2C12 myotubes transfected with si‐circSnd1‐1 and si‐circSnd1‐2 in tumour necrosis factor alpha (TNF‐α)–induced muscle atrophy model. (D) Left: Performing immunofluorescent staining and measuring the diameter of C2C12 myotubes transfected with si‐circSnd1‐1 and si‐circSnd1‐2 in angiotensin II (Ang II)–induced muscle atrophy model (*n* = 6 per group; scale bar: 50 μm). Right: MuRF‐1 and Atrogin‐1 (*n* = 6 per group) mRNA expressions in C2C12 myotubes transfected with si‐circSnd1‐1 and si‐circSnd1‐2 in angiotensin II (Ang II)–induced muscle atrophy model. Two‐way ANOVA with Tukey test was used (A‐D). *p < 0.05; **p < 0.01; ***p < 0.001. Data are represented as mean ± SD.

To investigate the potential protective role of silencing circSnd1 in muscle atrophy, we first explored whether circSnd1 knockdown protected against multiple types of muscle atrophy in vitro. Three small interfering RNAs (siRNAs) were used to target the back‐splice sequence of circSnd1, thereby restricting circSnd1 expression and without affecting the Snd1 mRNA expression in C2C12 myotube cells (Figure 4A). Two specific circSnd1 siRNAs were chosen to transfect into myotube cells and then subsequently induced muscle atrophy by Dex, TNF‐α and Ang II, separately. Myotube diameter and atrogene expression were analysed to determine the influence on muscle atrophy. We found that circSnd1 knockdown increased myotube diameter and suppressed atrogene expression (Figure 4B–D). This indicated that inhibition of circSnd1 attenuated muscle atrophy in vitro. Meanwhile, we also found circSnd1 knockdown increased myotube diameter, suppressed atrogene expression and decreased SA‐β‐gal activity in H_2_O_2_‐induced muscle atrophy and myotube senescence (Figure S6).

**FIGURE 5:**
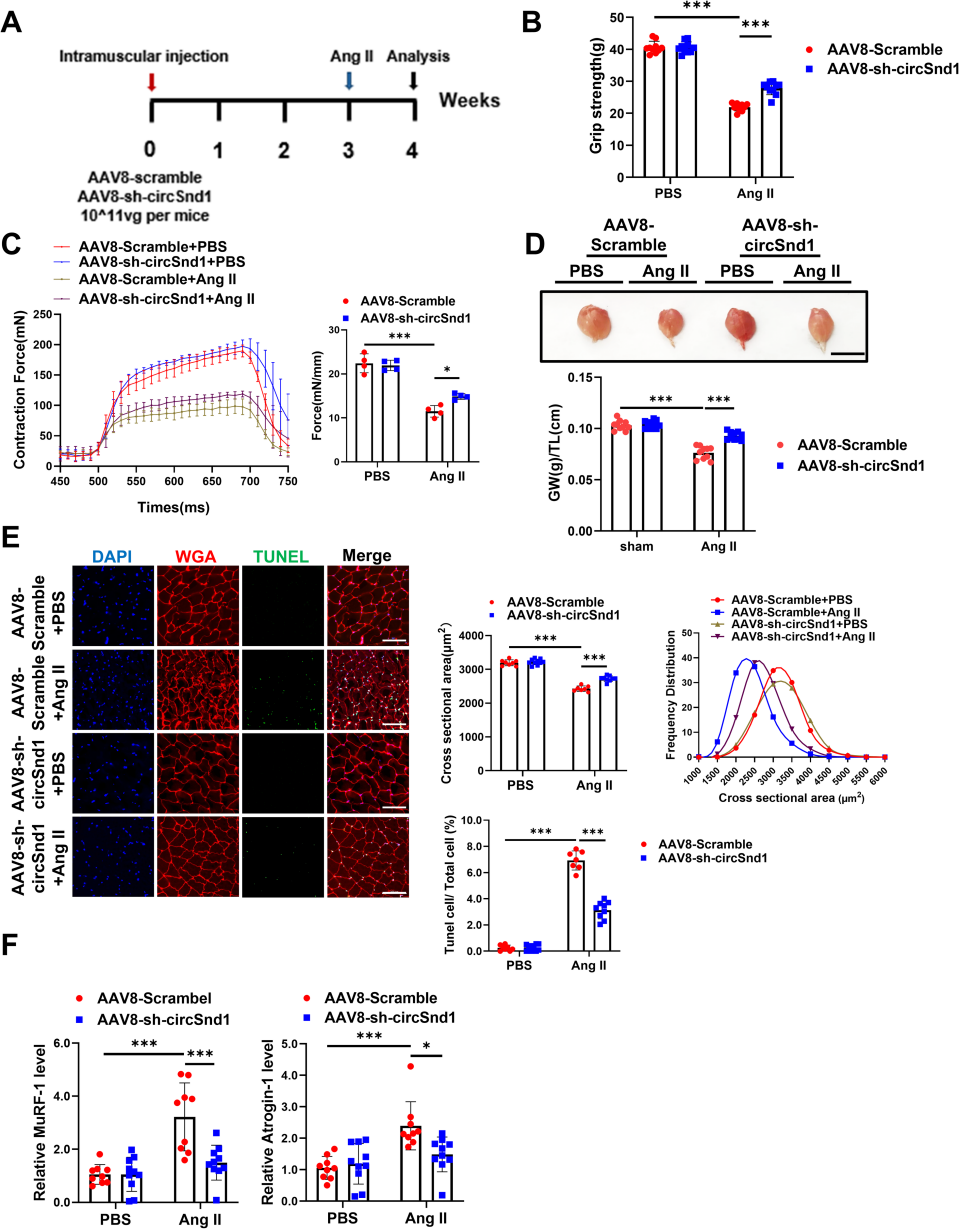
Inhibition of circSnd1 prevents angiotensin II (Ang II)–induced muscle atrophy in vivo. (A) Experimental flowchart. (B) Comparing the grip strength of mice injected with AAV8‐sh‐circSnd1 to those injected with AAV8‐Scramble in AngII‐induced muscle atrophy (*n* = 10 per group). (C) Comparing the muscle tetanic contraction of mice injected with AAV8‐sh‐circSnd1 to those injected with AAV8‐Scramble in AngII‐induced muscle atrophy in EDL muscle (*n* = 4 per group). (D) The mouse gastrocnemius muscle morphology and the ratio of gastrocnemius weight (GW) to tibia length (TL) of mice after the injection of AAV8‐sh‐circSnd1 compared to AAV8‐Scramble under the treatment of AngII (*n* = 10 per group; scale bar, 1 cm). (E) WGA staining and TUNEL staining detect CSA and cell apoptosis of gastrocnemius muscle in mice injected with AAV8‐sh‐circSnd1 in angiotensin II (Ang II)–induced muscle atrophy (*n* = 8, 10, 7, 9; scale bar: 100 μm). (F) mRNA level of Atrogin‐1 and MuRF‐1 expression in mouse gastrocnemius muscle after the injection of AAV8‐sh‐circSnd1 compared to AAV8‐Scramble under the treatment of Ang II (*n* = 9, 10, 9, 10). Two‐way ANOVA with Tukey test was used (B–F). *p < 0.05; ***p < 0.001. Data are represented as mean ± SD.

To further investigate the potential protective effect of circSnd1 silencing in muscle atrophy in vivo, AAV8‐mediated shRNA was used to reduce circSnd1 expression in mice. The renin–angiotensin system (RAS) contributes to cellular and organ ageing primarily through the overproduction of reactive oxygen species, activating the ACE/Ang II/Ang II Type 1 receptor pathway [17, 18]. Thus, we introduced Ang II‐induced muscle atrophy to mimic the ageing associated muscle atrophy and evaluated the function of circSnd1 in muscle atrophy and muscle ageing. Adult male mice received a single intramuscular injection of AAV8‐circSnd1 or AAV8‐scramble, which was followed by Ang II perfusion (1 week) after 3 weeks post–AAV8 injection (Figure 5A and Figure S7A). The grip strength and tetanic forces were enhanced after the injection of sh‐circSnd1 in Ang II‐treated mice (Figure 5B,C). The gastrocnemius muscle weight was decreased in Ang II‐treated mice; however, muscle weight increased after circSnd1 inhibition (Figure 5D). To strengthen these findings, WGA staining on muscle cryosections revealed that reduced CSA of myofibre during Ang II was prevented in circSnd1 knockdown mice (Figure 5E). In addition, qRT‐PCR for atrogenes showed that circSnd1 knockdown repressed the expression of Atrogin‐1 and MuRF‐1 in Ang II‐treated mice (Figure 5F). In addition, the ratio of muscle cell death was also decreased after the injection of sh‐circSnd1 in Ang II‐treated GA muscle (Figure 5E and Figure S7B). Moreover, the reboot of AKT/FOXO3A/mTOR signalling emerged after the injection of sh‐circSnd1 in Ang II‐treated GA muscle (Figure S7C). In addition, circSnd1 inhibition prevented a fibre‐type shift in GA muscle from the fibres Type IIa to Type IIb (Figure S7D). These findings suggested that Ang II‐induced muscle atrophy was partially prevented by circSnd1 suppression.

In addition to the AngII‐induced muscle atrophy model, we also explored the potential protective role of silencing circSnd1 in Den and Imo‐induced muscle atrophy in vivo. circSnd1 knockdown partially rescues Den and Imo muscle atrophy by increasing muscle mass and myofibre CSA, reducing Atrogin‐1 and MuRF‐1 expression and reactivating the AKT/FOXO3A/mTOR pathway (Figures S8 and S9). CircSnd1 inhibition did not alter the myofibre composition in these muscle atrophy models (Figure S10). Overall, these results suggested that silencing of circSnd1 protected muscle atrophy in vivo.

### 3.4 CircSnd1 Interacts With the EEF1A1 Protein and Promotes Its Stability

**FIGURE 6:**
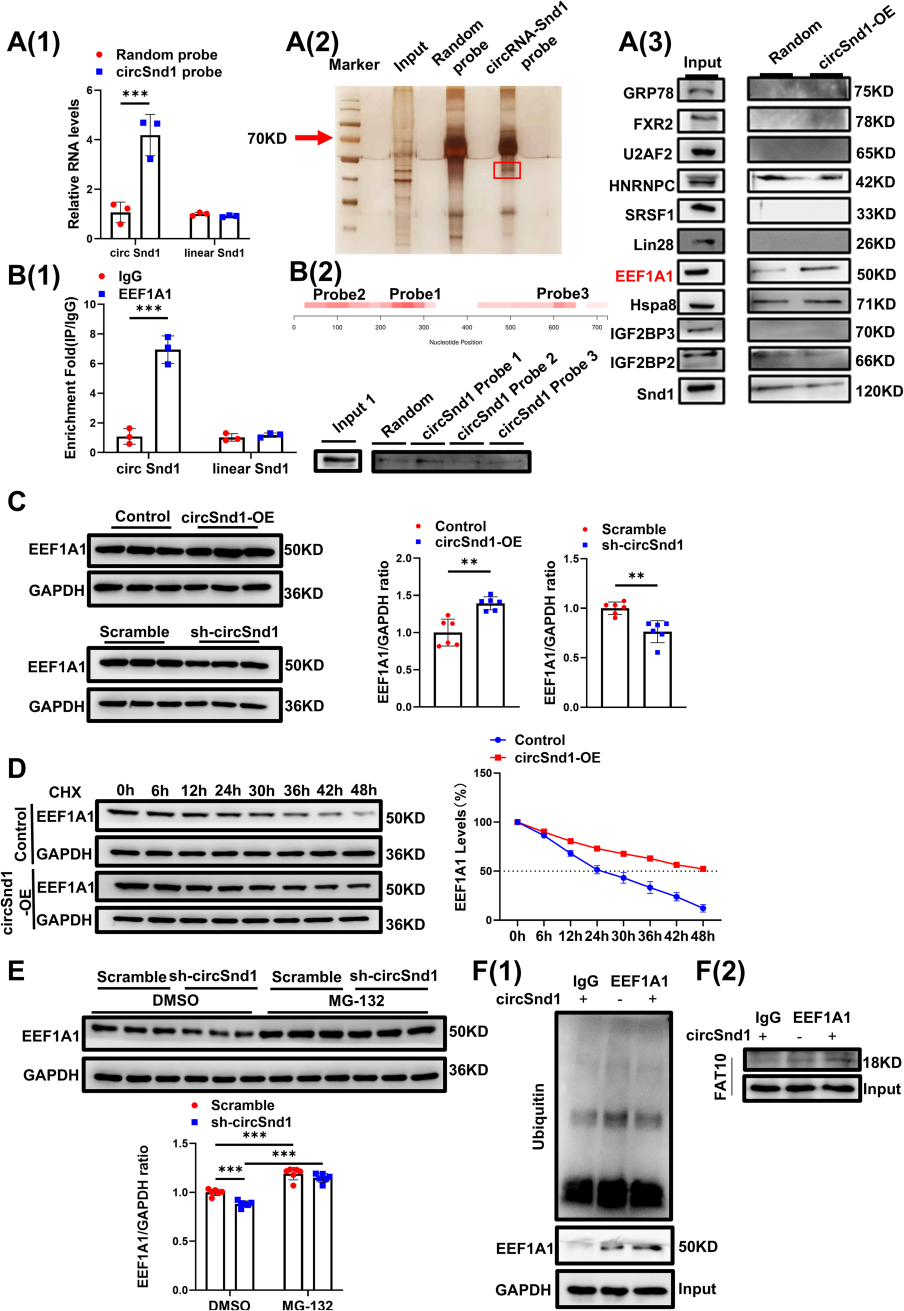
CircSnd1 interacts with EEF1A1 protein. (A) (1) Precipitation obtained by RNA pulldown assay using the circSnd1 probe (*n* = 3 per group). The enrichment of circSnd1 was verified by qRT‐PCR. (2) circSnd1 RNA Pulldown results. (3) Identify the target proteins after conducting the circSnd1 probe pulldown assay. (B) (1) RIP assay to analysis the enrichment of circSnd1 by EEF1A1 antibody (*n* = 3 per group). (2) RNA pulldown results from different region probes. (C) Protein level of EEF1A1 in C2C12 myotubes transfected with circSnd1 overexpression plasmid or circSnd1 shRNA plasmid (*n* = 6 per group). (D) Western blot analysis of EEF1A1 expression in C2C12 myotubes transfected with circSnd1 overexpression plasmid after CHX treatment. (E) Western blot analysis of EEF1A1 expression in C2C12 myotubes transfected with circSnd1 knockdown plasmid after treatment with MG‐132 (*n* = 6 per group). (F) (1) IP assay analysis for the level of protein ubiquitination of EEF1A1 in C2C12 myotubes after transfected with circSnd1 overexpression plasmid. (2) IP assay analysis for FAT10 protein expression in C2C12 myotubes after transfected with circSnd1 overexpression plasmid. An unpaired, two‐tailed Student's *t*‐test was used in (A–C). Two‐way ANOVA with Tukey test was performed in (E). **p < 0.01; ***p < 0.001. Data are represented as mean ± SD.

To reveal the mechanism by which circSnd1 promoted muscle atrophy, RNA pulldown assay was performed to identify the potential RBP, followed by silver staining (Figure 6A(1) and (2)). EEF1A1 was identified as one particularly abundant RBP according to mass spectrometer (MS) analysis (Figure 6A(3)). Furthermore, the association between EEF1A1 and circSnd1 was further validated by RNA immunoprecipitation (RIP) using an EEF1A1 antibody (Figure 6B(1)). To further clarify the specific functional domain between circSnd1 and EEF1A1, we performed in silico analyses via CatRAPID. Our results showed that three possible regions (nucleotides 227–276; nucleotides 77–124; and nucleotides 601–624) of circSnd1 had a high potential binding efficiency to EEF1A1 (Figure 6B(2), upper). Next, we performed the RNA pulldown assay in C2C12 myotube cells to further validate the association and transcribed the anticipated interaction areas (probe‐1:227–276, probe‐2:77–124 and probe‐3:601–624) of circSnd1 in vitro. The region of nucleotides 227–276 was the major binding domain between EEF1A1 and circSnd1 (Figure 6B(2), below). We also found circSnd1 positively regulates the protein level of EEF1A1 in cellular and animal levels (Figure 6C and Figure S11A–D). In addition, EEF1A1 was upregulated in multiple types of muscle atrophy models in vitro and in vivo (Figure S11E,F). These data suggested circSnd1 could bind with EEF1A1 and promote the protein level of EEF1A1 in skeletal muscle cells.

To further investigate how circSnd1 promotes the protein level of EEF1A1 after binding with each other, we analysed the protein stability of EEF1A1 by using the protein synthesis inhibitor cycloheximide (CHX). Endogenous EEF1A1 protein degraded in the presence of CHX, with the half‐life of EEF1A1 protein being about 24 h, whereas overexpressed circSnd1 could improve the stability of EEF1A1 with a half‐life of 48 h (Figure 6D). To test whether circSnd1‐dependent stabilization of EEF1A1 is due to the UPS, C2C12 myotube cells expressing circSnd1 shRNA were treated with the proteasome inhibitor MG132. We found that circSnd1 knockdown inhibited the protein level of EEF1A1, but MG132 protected EEF1A1 from degradation in circSnd1 knockdown (Figure 6E). To further confirm that, we then performed an IP assay to analyse the regulation of circSnd1 on EEF1A1 ubiquitination and degradation. We found that circSnd1 could repress ubiquitination of EEF1A1 (Figure 6F(1)). Targeting proteins directly for proteasomal destruction, FAT10 is a ubiquitin‐like modifier that counteracts ubiquitination to sustain target protein expression [19, 20, 21]. FAT10 and ubiquitin showed a competitive relationship in binding to the same lysines on EEF1A1. This means that when FAT10 is overexpressed, Ub‐EEF1A1 levels drop and FAT10‐EEF1A1 levels rise, stabilizing the protein level of EEF1A1 [22]. Our IP assay with EEF1A1 antibody revealed that circSnd1 could promote the bind between FAT10 and EEF1A1 (Figure 6F(2)). These data suggested that circSnd1 promoted the binding between FAT10 and EEF1A1 and competed with ubiquitin for binding to EEF1A1. This subsequently decreased the ubiquitination of EEF1A1 to stabilize the protein level of EEF1A1 in skeletal muscle cells.

**FIGURE 7:**
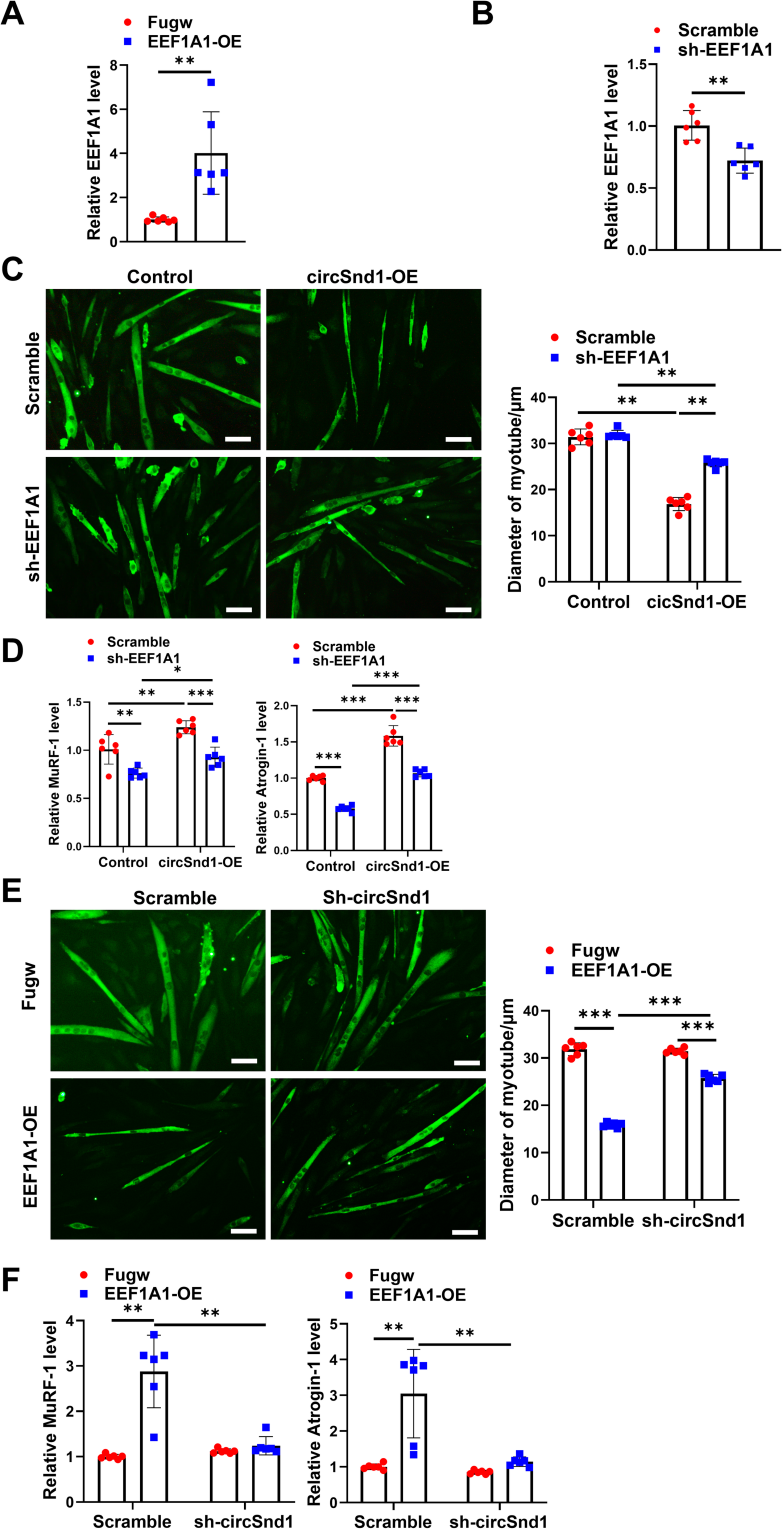
CircSnd1 binds to the EEF1A1 protein to promote muscle atrophy in vitro. (A,B) mRNA level of EEF1A1 in C2C12 myotubes transfected with EEF1A1 overexpression plasmid or EEF1A1 shRNA plasmid compared to control group (*n* = 6 per group). (C) Performing immunofluorescent staining and measuring the diameter of C2C12 myotubes transfected with circSnd1 overexpression and EEF1A1 shRNA plasmid (*n* = 6 per group; scale bar: 50 μm). (D) mRNA levels of MuRF‐1 and Atrogin‐1 in C2C12 myotubes transfected with circSnd1 overexpression and EEF1A1 shRNA plasmid (*n* = 6 per group). (E) Performing immunofluorescent staining and measuring the diameter of C2C12 myotubes transfected with EEF1A1 overexpression and circSnd1 shRNA plasmid (*n* = 6 per group; scale bar: 50 μm). (F) mRNA levels of MuRF‐1 and Atrogin‐1 in C2C12 myotubes transfected with EEF1A1 overexpression and circSnd1 shRNA plasmid (*n* = 6 per group). An unpaired, two‐tailed Student's *t*‐test was used (A,B), Two‐way ANOVA with Tukey test was performed (C–F). *p < 0.05; **p < 0.01; ***p < 0.001. Data are represented as mean ± SD.

To further investigate whether circSnd1‐induced muscle atrophy was mediated by EEF1A1, we simultaneously intervened circSnd1 and EEF1A1. EEF1A1 overexpression and shRNA plasmids were constructed to manipulate the expression of EEF1A1 (Figure 7A,B). The rescue experiment showed that inhibition of EEF1A1 could alleviate circSnd1 overexpression–induced muscle atrophy (Figure 7C,D). In addition, overexpression of EEF1A1 in C2C12 myotubes could promote muscle atrophy (Figure S12). Moreover, the rescue experiment showed that EEF1A1 could promote muscle atrophy, although knockdown of circSnd1 could rescue that (Figure 7E,F). This suggested that EEF1A1 was involved in the pathogenesis of circSnd1‐induced muscle atrophy in vitro.

### 3.5 RNA Binding Protein EIF4A3 Regulates the Expression of circSnd1 in Muscle Atrophy

**FIGURE 8:**
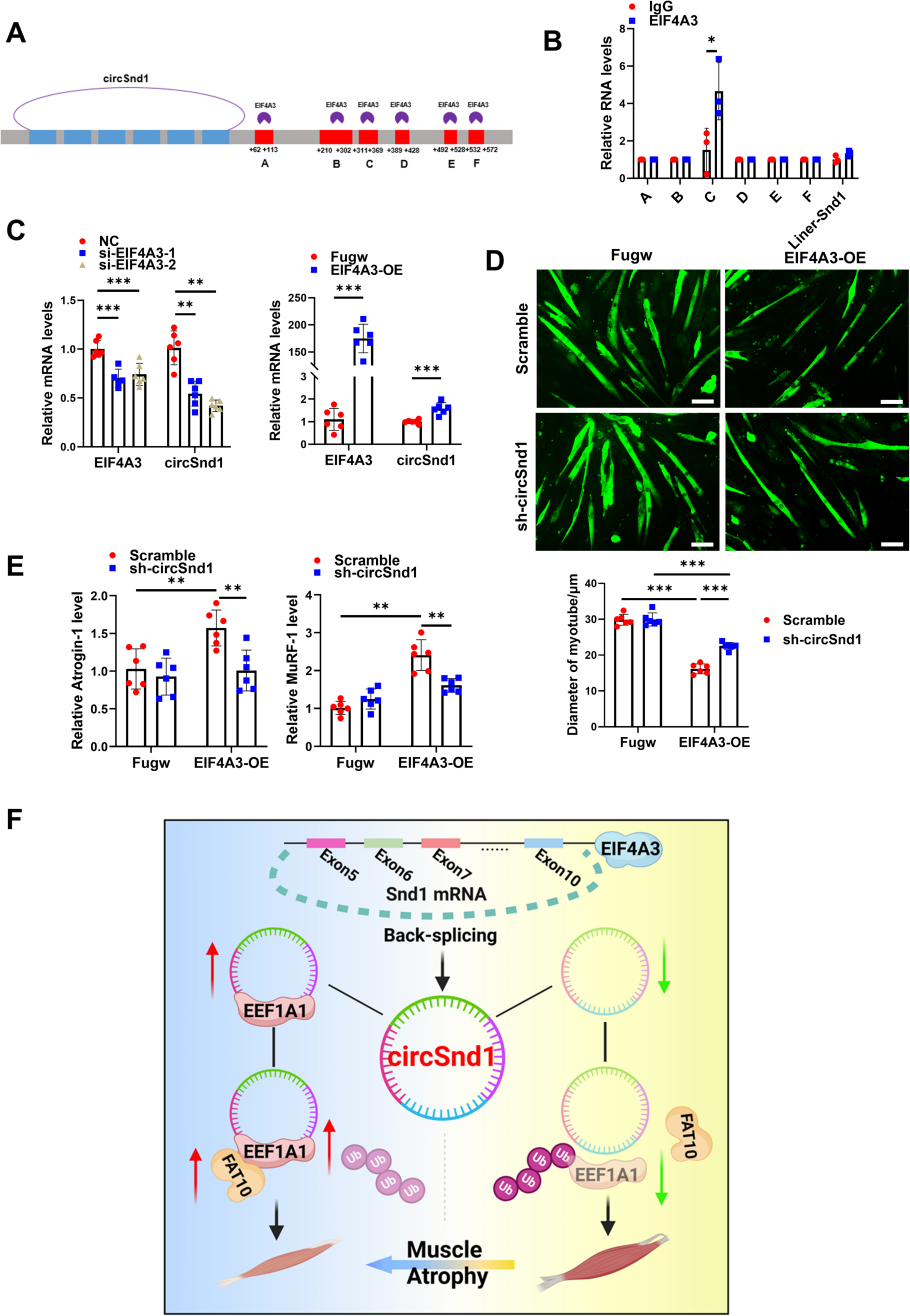
EIF4A3‐induced circSnd1 expression in muscle atrophy. (A) The binding sites between EIF4A3 and circSnd1 flanking sequences. (B) RIP assay to analysis the enrichment of circSnd1 flanking sequences by EIF4A3 antibody (*n* = 3 per group). (C) RNA levels of EIF4A3 and circSnd1 in C2C12 myotube cells treated with EIF4A3 overexpression plasmid and si‐EIF4A3 compared to control (*n* = 6 per group). (D) Immunofluorescent staining and quantification of the diameter in C2C12 myotube cells treated with EIF4A3 overexpression plasmid and circSnd1 shRNA plasmid (*n* = 6 per group; scale bar: 50 μm). (E) mRNA levels of Atrogin‐1 and MuRF‐1 in C2C12 myotube cells treated with EIF4A3 overexpression plasmid and circSnd1 shRNA plasmid (*n* = 6 per group). (F) Illustration of the mechanisms of circSnd1 in muscle atrophy. The figure is created with BioRender (Biorender.com). An unpaired, two‐tailed Student's *t*‐test was used in (B,C), Two‐way ANOVA with Tukey test was performed in (D and E). *p < 0.05; **p < 0.01; ***p < 0.001. Data are represented as mean ± SD.

To clarify the regulatory upstream mechanism underlying circSnd1 formation, we analysed the RBP that could bind to the genomic sequencing of the flanking intron regions and affect circSnd1 circularization. circInteractome databases (https://circinteractome.nia.nih.gov/index.html) showed that EIF4A3 had six potential binding sites in the downstream of circSnd1 RNA transcript (Figure 8A and Figure S13A). EIF4A3 RIP assay indicated that EIF4A3 could bind to the region C (Figure 8B). Furthermore, in vitro experiments revealed that EIF4A3 overexpression significantly upregulated circSnd1, whereas EIF4A3 knockdown markedly attenuated its expression in muscle cells (Figure 8C and Figure S13B). Furthermore, EIF4A3 was increased in muscle atrophy models (Figure S13C). These results indicated that EIF4A3 could bind to the flanking sequences of circSnd1 and promote circSnd1 expression.

To further clarify the functional interaction between EIF4A3 and circSnd1, we performed the rescue experiment in vitro by modulating the EIF4A3 and circSnd1 expression independently or respectively. We found that EIF4A3 overexpression induced muscle atrophy, whereas knockdown of circSnd1 at the same time could reverse the muscle atrophy phenotype (Figure 8D,E). Similarly, repression of EIF4A3 could attenuate muscle atrophy in vitro and circSnd1 overexpression abolished the protective effect mediated by EIF4A3 inhibition in muscle atrophy (Figure S13D,E). In summary, these data suggested that EIF4A3 promoted the expression of circSnd1 during muscle atrophy in vitro.

## Discussion

With the development of next generation RNA sequencing technology as well as the advanced bioinformatics algorithms, thousands of circRNAs have been identified as the regulators for physiological or pathological processes. Therefore, seeking the disease‐relevant circRNAs may be an effective method for exploring therapeutic targets. In the present study, we found that circSnd1 is a common target for multiple types of muscle atrophy. EIF4A3 induced circSnd1 expression and further promoted muscle atrophy. Mechanistically, circSnd1 enhanced the binding between FAT10 and EEF1A1. FAT10 competed with ubiquitin for binding with EEF1A1 and stabilized the protein level of EEF1A1 in muscle cells, leading to the atrophy (Figure 8F).

Despite the rapidly increasing number of circRNAs functionally investigated so far, their effects on muscle atrophy still are limited. Our findings provide a comprehensive view of circSnd1 function and mechanism in muscle atrophy. By circRNA omics and PCR screen, we identified circSnd1 as a conserved regulator in muscle atrophy under different stimulation in humans and mice. circSnd1 was shown to be upregulated in a number of muscle atrophy models. We therefore investigated the role of circSnd1 in C2C12 myotube cells and mice and observed that circSnd1 overexpression significantly promoted muscle atrophy. In contrast, inhibition of circSnd1 alleviated the muscle mass and muscle function in different types of muscle atrophy in vivo. Especially, Snd1 (host gene of circSnd1) linear mRNA expression level was not changed in muscle atrophy models (Figure S1C–H). This phenomenon is mainly due to the back‐splicing of circRNA from pre‐mRNA and largely depended on a post‐transcriptional regulatory model [23]. CircRNA formation is controlled by *cis*‐regulation elements, which is different from the transcription of mRNA. However, we also found circSnd1 was ubiquitously expressed in different tissues and the muscle system. Therefore, further study needs to further clarify whether circSnd1 participated in atrophy in other tissues.

To further study the mechanism for circSnd1 upregulation in muscle atrophy, we focused on the splicing factor EIF4A3. As a core helicase component of the exon junction complex, EIF4A3 regulates RNA metabolism by participating in RNA splicing, mRNA trafficking and affecting the downstream events [24, 25]. In particular, EIF4A3 can enhance the biogenesis of some circRNAs by interacting with the flanking sequence of pre‐circRNAs, such as circSEPT9 [S19], circASAP1 [S20], circPRKAR1B [S21] and circARHGAP29 [S22]. We found that EIF4A3 promoted the circularization and expression of circSnd1 by binding the downstream flanking sequence of circSnd1. Moreover, EIF4A3 was upregulated in different muscle atrophy models, and EIF4A3 promoted muscle atrophy [26]. These results indicated that EIF4A3 might be a key splicing factor that facilitates the function of circRNAs in muscle atrophy.

Sponging miRNAs has been the focus point in many prior studies aiming to underlie the mechanism of circRNAs [9]. For example, circIGF1R regulates myogenesis by sponging miR‐16 [27]. Numerous experimental studies have demonstrated that long non‐coding RNAs, pseudogene transcripts, circRNAs, viral RNAs and protein‐coding transcripts can function as competing endogenous RNAs (ceRNAs) [28]. However, for a circRNA to effectively serve as a ceRNA, it must possess a sufficient number of miRNA binding sites to significantly impact the repression of miRNA targets [29]. In our study, we observed that circSNDI interacts with multiple miRNAs but has a limited number of miRNA sponging sites (less than two for most miRNAs) (Figure S14), raising doubts about its efficacy as a miRNA regulator. Currently, some studies reported that circRNA could interact with RBPs in skeletal muscle [30]. Our research revealed that circSnd1 binds to EEF1A1 and facilitates the FAT10–EEF1A1 complex, thus enhancing the stability of EEF1A1 and inhibiting the ubiquitination of EEF1A1 in muscle atrophy.

EEF1A1 belongs to the family of elongation factor 1α gene family. The two actively transcribed genes, EEF1A and EEF1A2, make up the eEFIA1 gene family [31]. They are essential to the process that converts mRNAs into proteins. EEF1A1 is one of the important regulators of various biological processes, such as cell cycle, cell growth and cell death [S23, S24]. However, the expression of eEF1A1 and eEF1A2 is generally considered to be mutually exclusive. In skeletal muscle, eEF1A1 is predominantly expressed in the embryonic, postnatal and ageing stages, whereas eEF1A2 transcription increases and takes over the eEF1A1‐specific function for protein synthesis in adult muscle [S25, 32]. In addition, the functions of EEF1A1 and EEF1A2 are not the same in muscle. EEF1A2 is overexpressed and selected over EEF1A1 in healthy muscle, whereas in stress models, eEF1A1 expression increased and was associated with proteolysis, apoptosis, and catabolism [33, 34, 35]. A previous study showed that eEF1A1 was upregulated in denervation‐induced muscle atrophy in the old rat [36]. The high expression of eEF1A1 was found in many muscle atrophy models in the current study. These findings indicated that eEF1A1 is the common regulator for different types of muscle atrophy. Moreover, EEF1A1 could trigger myotube muscle atrophy. As an ubiquitin‐like protein (UBL), human HLA‐F adjacent transcript 10 (FAT10) could compete with Ub to bind with the same lysine of the substrate, leading to the formation of FAT10–substrate complexes and ubiquitination of antagonized substrate [37, 38]. Notably, eEF1A1 was a binding protein that was unique to FAT10 [39]. In our study, we found that circSnd1 could enhance the binding efficiency between FAT10 and EEF1A1 and compete with ubiquitin for binding to EEF1A1, leading to the less ubiquitination of EEF1A1 and maintaining the stabilization of EEF1A1 in skeletal muscle cells. In addition, circSnd1 did not change the expression of EEF1A2 in skeletal muscle cells (Figure S15).

In conclusion, we clarified that circSnd1 was upregulated during muscle atrophy. High levels of circSnd1 could induce muscle atrophy, and knockdown of circSnd1 indicated a protective effect on multi‐factor‐induced muscle atrophy. EIF4A3‐induced circSnd1 mediated muscle atrophy by promoting the stability of EEF1A1 via recruiting FAT10. Thus, circSnd1 could serve as a potential therapeutic target for muscle atrophy caused by multiple factors.

## Funding

This work was supported by the grants from National Key R&D Program of China (2020YFA0803800 to J.L.), National Natural Science Foundation of China (82020108002 and 82225005 to J.X.; 82471618 and 82271623 to J.L.; 82401851 to T.Y.; 82400423 to D.L.; 825722922 to Y.Z.), Science and Technology Commission of Shanghai Municipality (23410750100 to J.X.; 22010500200 to J.L.; 23ZR1420500 to Y.Z.), the Shanghai Rising‐Star Program (23QA1403600 to J.L.) and the Shanghai Pujiang Program (23PJ1412200 to D.L.).

## Conflicts of Interest

The authors declare no conflicts of interest.

## Supporting information


**Figure S1:** circDdb1 (circBase ID: mmu_circ_0013252) is conserved between human (circBase ID: hsa_circ_0082113) and mouse species. (A) Sequence alignment of *mmu*_circDdb1 and *hsa*_circSND1. (B) Analysing the cirSND1 by performing gel electrophoresis and Sanger sequencing of the back‐splice junction.
**Figure S2:** The circSnd1 and Snd1 content in muscle atrophy. (A,B) Distribution of circSnd1 in different tissues and organs (*n* = 3 per group). (C–E) mRNA level of Snd1 in gastrocnemius muscle atrophy model including the treatment of denervation (Den) and immobilization (Imo) as well as angiotensin II (AngII) (*n* = 6 per group). (F–H) mRNA level of Snd1 in C2C12 myotubes with the treatment of dexamethasone (Dex), tumour necrosis factor alpha (TNF‐α) and AngII (*n* = 6 per group). (I) RNA of circSnd1 in different types of skeletal muscle (Type I: soleus; Type II: gastrocnemius; combination of Type II and Type I: tibialis anterior) in muscle atrophy model induced by immobilization (*n* = 3 per group). An unpaired, two‐tailed Student's *t*‐test was used for comparisons between two groups. **p < 0.01. Data are represented as mean ± SD.
**Figure S3:** circSnd1 promotes muscle atrophy in vitro. (A) mRNA level of UPS‐related genes in circSnd1‐OE (stimulated by circSnd1 overexpression plasmid) C2C12 myotubes compared to control group (*n* = 6 per group). (B) mRNA level of autophagy‐related genes in circSnd1‐OE C2C12 myotubes compared to control group (*n* = 6 per group). (C) Protein level of AKT, FOXO3A, mTOR, P70S6K and4EBP1 expression in circSnd1‐OE C2C12 myotubes compared to control group (*n* = 6 per group). (D) The content of mitochondrial DNA (mtDNA) normalized by genomic DNA in circSnd1‐OE C2C12 myotubes compared to control group (*n* = 6 per group). An unpaired, two‐tailed Student's *t*‐test was used for comparisons between two groups. **p < 0.01; ***p < 0.001. Data are represented as mean ± SD.
**Figure S4:** CircSND1 promotes muscle atrophy in human myotube in vitro. (A) RNA levels of circSND1 expression in circSND1‐OE plasmid treated human myotubes compared to control group (*n* = 6 per group). (B) Representative images and statistical analysis of human myotubes transfected with circSND1‐OE and control plasmid (*n* = 6; scale bar: 50 μm). (C) Expression levels of MuRF‐1 and Atrogin‐1 mRNA levels in human myotube transfected with circSND1‐OE and controls plasmid (*n* = 6). An unpaired, two‐tailed Student's *t*‐test was used for comparisons between two groups. **p < 0.01; ***p < 0.001. Data are represented as mean ± SD.
**Figure S5:** circSnd1 promotes muscle atrophy in vivo. (A) mRNA level of UPS‐related genes in mouse gastrocnemius muscle treated with circSnd1‐OE (circSnd1 overexpression) AAV8 (AAV8‐circSnd1‐OE) compared to the control AAV8 (*n* = 10, 8). (B) mRNA level of autophagy‐related genes in mouse gastrocnemius muscle treated with AAV8‐ circSnd1‐OE compared to control AAV8 (*n* = 10, 8). (C) Protein level of AKT, FOXO3A, mTOR, P70S6K and4EBP1 in mouse gastrocnemius muscle with AAV8‐circSnd1‐OE injection compared to AAV8‐control (*n* = 6 per group). (D) The types of gastrocnemius fibres in mice injected with AAV8‐circSnd1‐OE and AAV8‐control were detected by immunofluorescence staining (*n* = 3 per group; scale bar: 100 μm). An unpaired, two‐tailed Student's *t*‐test was used for comparisons between two groups. *p < 0.05; **p < 0.01; ***p < 0.001. Data are represented as mean ± SD.
**Figure S6:** Inhibition of circSnd1 expression prevents H_2_O_2_‐induced muscle atrophy and muscle ageing. (A) Immunofluorescent staining and the diameter of C2C12 myotubes transfected with si‐circSnd1 in H_2_O_2_‐induced muscle atrophy model (*n* = 6 per group; scale bar: 50 μm). (B) mRNA levels of MuRF‐1 and Atrogin‐1 (*n* = 6 per group) in C2C12 myotubes transfected with si‐circSnd1 H_2_O_2_‐induced muscle atrophy model. (C) SA‐β‐gal staining in C2C12 myotubes transfected with si‐circSnd1in H_2_O_2_‐induced muscle atrophy model (*n* = 5 per group; scale bar: 50 μm). Two‐way ANOVA with Tukey test was performed. *p < 0.05; **p < 0.01; ***p < 0.001. Data are represented as mean ± SD.
**Figure S7:** Inhibition of circSnd1 prevents angiotensin II (Ang II) induced muscle atrophy in vivo.(A) RNA level of circSnd1 expression in mouse gastrocnemius muscle after the injection of AAV8‐sh‐circSnd1 compared to AAV8‐Scramble under the treatment of angiotensin II (AngII) (*n* = 9, 10, 9,10). (B) Protein level of Bax, Bcl2 and Caspase3 expression in mice injected with AAV8‐sh‐circSnd1 compared to AAV8‐Scramble under the treatment of Ang II (*n* = 6 per group). (C) Protein level of AKT, FOXO3A, mTOR, P70S6K and 4EBP1 expression in mice injected with AAV8‐sh‐circSnd1compared to AAV8‐Scramble under the treatment of Ang II (*n* = 6 per group). (D) The types of gastrocnemius fibres in muscle of mice injected with AAV8‐sh‐circSnd1 and AAV8‐Scramble were detected by immunofluorescence staining (*n* = 3; scale bar: 100 μm). Two‐way ANOVA with Tukey test was performed. *, *p* < 0.05; **, *p* < 0.01; ***, *p* < 0.001. Data are represented as mean ± SD.
**Figure S8:** Inhibition of circSnd1 prevents denervation‐induced muscle atrophy in vivo. (A) Schematic diagram of experimental design. (B) RNA level of circSnd1 expression in mouse gastrocnemius muscle after the injection of AAV8‐sh‐circSnd1 compared to AAV8‐Scrambleunder the treatment of denervation (Den) (*n* = 10, 10, 10, 8). (C) Gastrocnemius muscle morphology and the ratio of gastrocnemius weight (GW) to tibia length (TL) in mice with AAV8‐sh‐circSnd1 injection compared to AAV8‐Scramble under the treatment of denervation (Den) (*n* = 10, 10, 10, 10; scale bar, 1 cm). (D) WGA staining for myofibre in mice with AAV8‐sh‐circSnd1 injection compared to AAV8‐Scramble under the treatment of denervation (Den) (*n* = 9, 8, 9, 9; scale bar: 100 μm). (E) mRNA levels of MuRF‐1and Atrogin‐1 expression in mice with AAV8‐sh‐circSnd1 injection compared to AAV8‐Scramble under the treatment of denervation (Den) (*n* = 10, 10, 10, 8). (F) Protein level of AKT, FOXO3A, mTOR, P70S6K and 4EBP1 expression in mice with AAV8‐sh‐circSnd1 injection compared to AAV8‐Scramble under the treatment of denervation (Den) (*n* = 6 per group). Two‐way ANOVA with Tukey test was performed (B–F). *p < 0.05; **p < 0.01; ***p < 0.001. Data were represented as mean ± SD.
**Figure S9:** Inhibition of circSnd1 prevents immobilization‐induced muscle atrophy in vivo. (A) Schematic diagram of experimental design. (B) RNA level of circSnd1 expression in mouse gastrocnemius muscle after the injection of AAV8‐sh‐circSnd1 compared to AAV8‐Scramble under the treatment of immobilization (Imo) (*n* = 10, 10, 8, 10). (C) The mouse gastrocnemius muscle morphology and the ratio of gastrocnemius weight (GW) to tibia length (TL) of mice after the injection of AAV8‐sh‐circSnd1 compared to AAV8‐Scramble under the treatment of immobilization (Imo) (*n* = 10 per group; scale bar: 1 cm). (D) WGA staining for mouse myofibre with the injection of AAV8‐sh‐circSnd1 compared to AAV8‐Scramble under the treatment of immobilization (Imo) (*n* = 9, 10, 9, 9; scale bar: 100 μm). (E) mRNA level of MuRF‐1 and Atrogin‐1 expression in mouse gastrocnemius muscle after the injection of AAV8‐sh‐circSnd1 compared to AAV8‐Scramble under the treatment of immobilization (Imo) (*n* = 10, 10, 8, 10). (F) Protein level of AKT, FOXO3A, mTOR, P70S6K and 4EBP1 expression in mice injected with AAV8‐sh‐circSnd1compared to AAV8‐Scramble in muscle atrophy induced by immobilization (Imo) (*n* = 6 per group). Two‐way ANOVA with Tukey test was performed (B–F). **p < 0.01; ***p < 0.001. Data were represented as mean ± SD.
**Figure S10:** Inhibition of circSnd1 does not change the muscle fibre composition in denervation and immobilization induced muscle atrophy in vivo. The types of gastrocnemius fibres in muscle of mice injected with AAV8‐sh‐circSnd1 and AAV8‐Scramble in denervation (A) and immobilization (B) induced muscle atrophy, which were detected by immunofluorescence staining (*n* = 3; scale bar: 100 μm). Two‐way ANOVA with Tukey test was performed. Data are represented as mean ± SD.
**Figure S11:** EEF1A1 is upregulated in muscle atrophy.(A) Protein level of EEF1A1 expression in circSnd1‐OE (stimulated by circSnd1 overexpression plasmid) C2C12 myotubes compared to control group (*n* = 6 per group). (B) Protein level of EEF1A1 expression in mouse gastrocnemius muscle injected with AAV8‐sh‐circSnd1 compared to AAV8‐Scramble in Den‐induced muscle atrophy (*n* = 6 per group). (C) Protein level of EEF1A1 expression in mouse gastrocnemius muscle injected with AAV8‐sh‐circSnd1 compared to AAV8‐Scramble in AngII‐induced muscle atrophy (*n* = 6 per group). (D) Protein level of EEF1A1 expression in mouse gastrocnemius muscle injected with AAV8‐sh‐circSnd1 compared to AAV8‐Scramble in IMO‐induced muscle atrophy (*n* = 6 per group). (E) Protein level of EEF1A1 expression in C2C12 myotube treated with Dex and TNFα as well as AngII (*n* = 6 per group). (F) Protein level of EEF1A1 expression in mouse gastrocnemius muscle samples treated with Den and AngII as well as IMO (*n* = 6 per group). An unpaired, two‐tailed Student's *t*‐test was used in (A), (E) and (F). Two‐way ANOVA with Tukey test was performed in (B–D). *p < 0.05; **p < 0.01; ***p < 0.001. Data are represented as mean ± SD.
**Figure S12:** EEF1A1 promotes muscle atrophy in vitro. (A) mRNA levels of EEF1A1 expression in EEF1A1‐OE lentivirus‐treated C2C12 myotubes compared to control group (*n* = 6 per group). (B) Representative images and statistical analysis of C2C12 myotubes transfected with EEF1A1‐OE and control lentivirus (*n* = 6; scale bar: 50 μm). (C) Expression levels of MuRF‐1 and Atrogin‐1 mRNA levels in C2C12 myotube transfected with EEF1A1‐OE and controls lentivirus (*n* = 6). An unpaired, two‐tailed Student's *t*‐test was used for comparisons between two groups. **p < 0.01. Data are represented as mean ± SD.
**Figure S13:** EIF4A3 induces circSnd1 expression in muscle atrophy. (A) The RBP of flanking sequences of circSnd1 were predicted using CircInteractome databases. (B) Protein level of EEF1A1 expression in C2C12 myotube cells treated with EIF4A3 overexpression plasmid and si‐EIF4A3 compared to control (*n* = 6). (C) Protein level of EIF4A3 expression in mouse gastrocnemius muscle samples from Dex‐, TNFα‐ and AngII‐induced muscle atrophy model (*n* = 6 per group). (D) Immunofluorescent staining and quantification of the diameter of C2C12 myotubes transfected with circSnd1‐OE plasmid and si‐EIF4A3 under dexamethasone (Dex) treatment (*n* = 6 per group; scale bar: 50 μm). (E) mRNA level of MuRF‐1 and Atrogin‐1 expression (*n* = 6 per group) in C2C12 myotubes transfected with circSnd1‐OE plasmid and si‐EIF4A3 under dexamethasone (Dex) treatment (*n* = 6 per group). An unpaired, two‐tailed Student's *t*‐test was used in (B) and (C). Two‐way ANOVA with Tukey test was performed (D,E). **p < 0.01; ***p < 0.001. Data are represented as mean ± SD.
**Figure S14:** The number of binding sites for miRNAs in circSnd1. The binding between circSnd1 and miRNAs was predicted using CircInteractome databases.
**Figure S15:** circSnd1 does not change the EEF1A2 mRNA expression. Expression levels of circSnd1 and EEF1A2 RNA levels in C2C12 myotube transfected with circSnd1‐OE and controls plasmid (*n* = 6). An unpaired, two‐tailed Student's *t*‐test was used for comparisons between two groups. **p < 0.01. Data are represented as mean ± SD.
**Table S1:** Primer for RT‐qPCR.
