# Supplementary material for: EIF4A3‐Induced Circular RNA circSnd1 Promotes Muscle Atrophy and Muscle Ageing by Stabilizing EEF1A1
Source: J Cachexia Sarcopenia Muscle. 2026 Jan 29;17(1):e70210. doi: 10.1002/jcsm.70210 (PMC12855621; doi:10.1002/jcsm.70210)
Supplement: Supplementary file 1 — Figure S1: circDdb1 (circBase ID: mmu_circ_0013252) is conserved between human (circBase ID: hsa_circ_0082113) and mouse species. (A) Sequence alignment of mmu_circDdb1 and hsa_circSND1. (B) Analysing the cirSND1 by performing gel electrophoresis and Sanger sequencing of the back‐splice junction. Figure S2: The circSnd1 and Snd1 content in muscle atrophy. (A,B) Distribution of circSnd1 in different tissues and organs (n = 3 per group). (C–E) mRNA level of Snd1 in gastrocnemius muscle atrophy model including the treatment of denervation (Den) and immobilization (Imo) as well as angiotensin II (AngII) (n = 6 per group). (F–H) mRNA level of Snd1 in C2C12 myotubes with the treatment of dexamethasone (Dex), tumour necrosis factor alpha (TNF‐α) and AngII (n = 6 per group). (I) RNA of circSnd1 in different types of skeletal muscle (Type I: soleus; Type II: gastrocnemius; combination of Type II and Type I: tibialis anterior) in muscle atrophy model induced by immobilization (n = 3 per group). An unpaired, two‐tailed Student's t‐test was used for comparisons between two groups. **p < 0.01. Data are represented as mean ± SD. Figure S3: circSnd1 promotes muscle atrophy in vitro. (A) mRNA level of UPS‐related genes in circSnd1‐OE (stimulated by circSnd1 overexpression plasmid) C2C12 myotubes compared to control group (n = 6 per group). (B) mRNA level of autophagy‐related genes in circSnd1‐OE C2C12 myotubes compared to control group (n = 6 per group). (C) Protein level of AKT, FOXO3A, mTOR, P70S6K and4EBP1 expression in circSnd1‐OE C2C12 myotubes compared to control group (n = 6 per group). (D) The content of mitochondrial DNA (mtDNA) normalized by genomic DNA in circSnd1‐OE C2C12 myotubes compared to control group (n = 6 per group). An unpaired, two‐tailed Student's t‐test was used for comparisons between two groups. **p < 0.01; ***p < 0.001. Data are represented as mean ± SD. Figure S4: CircSND1 promotes muscle atrophy in human myotube in vitro. (A) RNA levels of circSND1 expr [file JCSM-17-e70210-s001.docx]

**Supplementary Information for
EIF4A3-induced circular RNA circSnd1 promotes muscle atrophy and muscle aging by stabilizing EEF1A1**

Jin Li^1,2,#^ , Bing Jin^1,2,#^ , Yuwei Yan^1,2,#^ , Yuying Chen^1,2^, Xiaohang Yin^1,2^, Xinyi Ren^1,2^, Qian Li^1,2^, Jingying Chen^1,2^, Siqi Wang^1,2^,Tingting Yang^1,2^, Yanan Zhang^1,2^, Qiumeng Nie^1,2^, Dongchao Lu^3^, Ming Wu^4^ ,Yan Yu^5^, Lei Chen^5^, Tarun Keswani^6^, Guoping Li^7^, Dragos Cretoiu^8,9^, T Scott Bowen^10^, Junjie Xiao^1,2,*^, Yongjun Zheng^11,*^

**1.** **Materials and methods**

**1.1 Cell culture and cellular atrophy models**

Mouse myoblast C2C12 cells and human renal epithelial cells 293T cells were cultured in DMEM medium (Corning, NY, USA) containing 10% fetal bovine serum (ExCell Bio, Shanghai, China) and 1% penicillin-streptomycin (KeyGen, Nanjing, China) at 37℃, 5% CO_2_ incubator.

Mature myotubes may be generated in 4–6 days by growing C2C12 in differentiation medium (DMEM with 2% horse serum and 1% penicillin and streptomycin) to induce myotube differentiation. Mature myotubes were treated with 50μM Dex (selleck, Shanghai, China), 100ng/ml TNF-α (Sino Biological, Beijing, China), 500 nM AngⅡ (Selleck, TX, USA) or 300μM H_2_O_2_ (Sigma, Darmstadt, Germany) for 24h to establish a cellular muscle atrophy model.

**Human skeletal muscle myoblasts were obtained from ZQXZbio (PRI-H-00152, shanghai ZhongQiao Xin Zhou Biotechnology co., Ltd, China). The cells were cultured in growth medium with 10% fetal bovine serum (ExCell Bio, China) and 1% penicillin-streptomycin (KeyGEN, China). The human myotube cells were obtained by switching differentiation medium (DMEM medium containing 2% horse serum, 1% penicillin-streptomycin and 10 μg/mL insulin) for maintaining 5 days. After that, the mature myotube human myotube was transfected with plasmids for 48h. And the treated cells were collected for further analyzing.**

**1.2 Cell transfection**

Following the detailed instructions provided by the manufacturer, Lipofectamine2000 reagent (Invitrogen, Carlsbad, CA, USA) was employed for cell transfection. siRNAs of circSnd1 were synthesized by RiboBio (Guangzhou, China) and the sequences used as follows: si-circSnd1-1:5’-AGACAACATACAGCCCAGA-3’; si-circSnd1-2:5’-CATACAGCCCAGAGCAGAA-3’; si-circSnd1-3: 5’-TACAGCCCAGAGCAGAACC-3’. The used dose of siRNA in our study was 50nM. The myotubes were transfected with circSnd1 siRNA to knockdown circSnd1 specifically, and then the muscle atrophy was induced 24h later.

**1.3 Quantitative reverse transcription polymerase chain reaction (qRT-PCR)**

Total RNA from cells and tissues was lysed and extracted using Trizol (TaKaRa, Kyoto, Japan) according to the standard experimental procedures. The concentration of RNA was measured using a NanoDrop™One/One C microvolume UV-Vis spectrophotometer (Thermo Fisher Scientific, Massachusetts, USA). RevertAid First-Strand cDNA Synthesis Kit (Thermo Fisher Scientific, Massachusetts, USA) was employed to perform cDNA synthesis from RNA samples. The quantitative experiment was conducted by ChanQ Universal SYBR qPCR Master Mix (Vazyme, Nanjing, China), and 18S rRNA was selected as the house keeping gene. In this study, we normalized the relative RNA levels using the 2^-ΔΔCq^ method. And the list of primers used were provided in Table S1.

**1.4 Western Blot**

Cell and tissue samples were lysed by preparing protein lysates containing 1% protease inhibitors (KeyGen, Nanjing, China) and 1% phosphatase inhibitors. The protein concentration was measured by BCA Protein Quantification kit (TaKara, Kyoto, Japan). 20μg proteins sample were isolated by SDS-PAGE electrophoresis and transferred to PVDF membranes (Pall Corporation, Port Washington, USA) followed by blocking with 5% skim milk at room temperature for 2h and washed with PBST. Then the blocked membranes were incubated with primary antibody overnight at 4°C, followed by an incubation with HRP-conjugated secondary antibody (1:1,0000, Jackson ImmunoResearch Laboratories Inc., USA) in the next day. Finally, the protein signals were displayed by High-sig ECL Western Blotting Substrate (Tanon, Tanon, China) and BIO-RAD chemiluminescence system.

The primary antibodies used include: GAPDH (Bioworld Technology #AP0066, Co., Ltd, Nanjing, China), p-mTOR (Ser-2448) (Abclonal #AP0115, Wuhan, China), mTOR (Abclonal #A11354, Wuhan, China), p-FOXO3A (Ser-253) (Abclonal #AP0684, Wuhan, China), FOXO3A (Abclonal #A9270, Wuhan, China), p-p70S6K (Abclonal #AP1059, Wuhan, China), p70S6K (Abclonal #A4898, Wuhan, China), p-AKT (Ser-473) (Cell Signaling Technology # 4060, Boston, USA), AKT (Abclonal #A18675, Wuhan, China), p-EIF-4EBP1 (Abclonal #AP0030, Wuhan, China), EIF4EBP1 (Abclonal #A1248, Wuhan, China), BAX (Abclonal #AP0030, Wuhan, China), BCL2 (Abclonal #A19693, Wuhan, China), Caspase3 (Abclonal #A2156, Wuhan, China), Cleaved caspase3 (Abclonal #A11021, Wuhan, China), LC3A/LC3B (Abclonal #A5618, Wuhan, China), P62 (Proteintech #18420-1-AP, Wuhan, China). EIF4A3 (Proteintech #17504-1-AP, Wuhan, China), EEF1A1 (Proteintech #67495-1-Ig, Wuhan, China), FAT10 (Abclonal #A9005, Wuhan, China)

**1.5 Immunofluorescence Staining**

The treated C2C12 myotubes were fixed with 4% PFA for 20 min and washed with PBS three times (5 min each time) at room temperature. After a 20-min treatment with 0.5% Trition X-100, the cells were washed three times with PBS. The cells were blocked at room temperature with 5% BSA for 2 h and incubated with Myosin sarcomere (MHC) primary antibody (1:100, DSHB, #MF-20-S) overnight at 4℃. Then, the cells labeled with the primary antibody were visualized by incubating them with the secondary antibody Alexa Fluor® 488 AffiniPure Goat Anti-Mouse IgG (H+L) (1:2000, Jackson, ImmunoResearch Laboratories Inc. USA) at room temperature for 2 h, while being protected from light. The nucleus was visualized by stainning with Hoechst (KeyGEN, Nanjing, China, 1:2000). **Fluorescence micrographs were acquired by using Leica DM i8 fluorescence microscope (Wetzlar, Germany) equipped with a 20× objective. Myotube diameters were quantified using Image J, with ≥50 myotubes analyzed per well. For each myotube, diameters were measured at three distinct positions perpendicular to the longitudinal axis, and the average value was recorded as the diameter of that individual myotube. Mean myotube diameter per group was subsequently calculated.**

**1.6 SA-β-gal staining**

SA-β-gal staining was performed by Cellular Senescence Detection Kit - SPiDER-βGal (Dojindo, Kumamoto, Japan) according to the instructions.

**1.7 Wheat germ agglutinin (WGA) staining**

Gastrocnemius muscle tissues were frozen in OCT and cut into 10 μm sections. The sections were fixed with 4% PFA for 15 min at room temperature and then incubated with WGA solution (1:100, Sigma-Aldrich, St. Louis, MI, USA) after rinsing with PBS three times. Then, staining with Hoechst solution (KeyGEN, Nanjing, China) for 20 min. The tissue sections were then washed for 3 times with PBS and sealed with 50% glycerin. Finally, a fluorescent microscope (Zeiss, Germany) was used to collect the pictures. We used the Image J program to measure the muscle fiber cross-sectional area, with a minimum of 500 muscle fibers analyzed per mouse.

**1.8 TUNEL staining**

The frozen muscle sections were removed from the -80°C refrigerator, placed in a wet box at room temperature for 10 min, and washed three times with 1×PBS. After that, the sections were incubated with 0.5% Triton X-100 at room temperature for 20 min. After blocking with 5% BSA was for 1 h, TUNEL staining was performed by using the DeadEnd fluorescent TUNEL system (Promega, Madison, WI, USA). Finally, Hoechst solution (KeyGEN, Nanjing, China) prepared in 1×PBS was incubated for 20 min at room temperature under dark conditions. The images were captured using a high resolution fluorescence microscope (Zeiss, Germany). The proportion of TUNEL-positive cells were analyzed using Image J software, and at least 500 muscle fibers were detected in each mouse.

**1.9 Pulldown assays**

Biotin-labeled oligonucleotide probes targeting circSnd1 junction sites were synthesized by Sangon Biotech (Shanghai, China). C2C12 cells were harvested after transfecting with circSnd1 overexpression plasmid for 48h. The cells were washed with pre-cooled 1×PBS and then resuspended in 1 ml of cell lysis buffer and incubated on ice for 10 min to fully lysate the cells. Cells were centrifuged at 12000xg for 10min at 4℃, 5% supernatant was taken as Input group and stored at -20℃. The remaining supernatant was equally divided into two groups, and 800μl of hybrid solution containing 10μg circSnd1 probe or Random probe was added and incubated at room temperature on a wheel overnight. Dynabeads^TM^ MyOne^TM^ Streptavidin T1(Invitrogen, CA, USA) was repeatedly washed with cell lysis buffer on a magnetic rack and incubated overnight by adding blocking solution (containing 50mg/ml BSA and Yeast tRNA) to a roller. The next day, the blocked magnetic beads were added to the cell supernatant containing the probe and incubated on a roller at room temperature for 3 h. After that, the beads were washed repeatedly on a magnetic rack with washing buffer and finally eluted with 100μl DEPC water. The RNA bound in the pull-down was subsequently detected using RT-qPCR. The recovered proteins in the complex were analyzed by western blotting or mass spectrometry (Genechem, Shanghai, China).

Following probe was used for the study:

probe-for-circSnd1: Biotin-5’-aaaCCTCTGTTGTATGTCGGGTCTCGTCTTGGC -3’.

**1.10 RNA Immunoprecipitation (RIP)**

C2C12 cells were collected after transfecting with circSnd1 overexpression plasmid for 48 h. Protease and RNase inhibitors-containing RIP lysis solution was added to the collected cells, and the mixture was spun at 4°C for 20 min. Following centrifugation at 12,000 rpm and 4°C for 30 min, the supernatant was collected. The supernatant was divided into two parts and incubated overnight with EEF1A1-specific antibody (Proteintech #67495-1-Ig, Wuhan, China), or EIF4A3(Proteintech #17504-1-AP, Wuhan, China) and negative control IgG (Santa cruz #sc-2025, CA, USA) at 4°C, respectively. On the next day, the antibodies containing cell supernatant was mixed with Dynabeads TM protein G and rotated for 3 h at 4 °C. RNeasy Mini Kit (Qiagen Inc., Valencia, CA, USA) was used to extract immunoprecipitated RNA following five washings with RIP lysis solution. The enrichment of circSnd1 was determined using a qRT-PCR assay.

**1.11 Co-Immunoprecipitation (Co-IP)**

C2C12 cells were infected with circSnd1 overexpressing plasmid and harvested after 72h. After washing with 1×PBS, cells were incubated in cell lysis buffer (Merck, Darmstadt, Germany) with protease inhibitor (Roche, Basel, Switzerland) for 2-3 h, followed by centrifugation at 12000 rpm for 30 min at 4°C. 10% of the cleared cell supernatant was collected as the Input control, and the remaining supernatant was divided into two groups and incubated with EEF1A1 antibody (Proteintech #67495-1-Ig, Wuhan, China) and control IgG antibody in a roller at 4°C overnight, respectively. Dynabeads Protein G (Thermo Fisher Scientific, Massachusetts, USA) (60 μl/ sample) was washed with 1×PBS for 5 times (2 min each time) and blocked overnight with 5% BSA (prepared with PBS). The next day, the cell lysate-antibody mixture incubated overnight was added with blocked Dynabeads Protein G, and incubated at 4℃ on a roller for 2-3 h. After washing with cell lysis buffer for 5 times (2 min each), the Dynabeads Protein G-binding protein was dissociated with 2×sample loading buffer (Thermo Fisher Scientific, Massachusetts, USA) and heated at 95°C for 10 min. The target protein expression in cell lysates was identified using immunoblotting.

**1.12 Muscle function test**

To assess the mouse grip strength, a digital Grip Strength Meter (Yiyan Technology Ltd., Shandong, China) was used to assess mouse grip strength. The operator placed the mouse on the digital grip strength meter for 3 min for adaptive adjustment. The mouse was allowed to grasp the metal pull rod on the meter and pull it horizontally and backward, and the digital grip strength meter showed the tensile value of the mouse. The effective value was recorded for each mouse three times, each time at an interval of 30 s-1 min, and the average values were calculated.

To measure the muscle tetanic contraction force, the EDL muscle was dissected and set up in an oxygenated physiological saline solution (Krebs Buffer) using standard methods. The tetanic contraction was then measured and controlled using dynamic muscle control software (Aurora Scientific, Aurora, Ontario, Canada) via direct electrical stimulations in accordance with a conventional experimental technique.

**1.13 Nuclear and cytoplasmic RNA separation**

C2C12 cells were harvested after digestion with Trypsin-EDTA (Gibco, Canada). After washing with 1×PBS, the cell precipitation was resuspended in 200μl of cell lysis buffer (10 mM pH=8.0 Tris, 1.5 mM MgCl2, 140 mM, NaCl, 0.5%NonidetP-40) for 5 min on ice. After centrifugation at 4°C for 3 min at 1500 rpm, the supernatant was transferred to a fresh EP tube and mixed with 1 mL Trizol, which was used to extract cytoplasmic RNA. And the cell precipitation was dissolved with a lysis buffer containing 1% Tween-40 and 0.5% Deoxycholic acid, and then washed with the buffer twice, and the purified nuclear precipitate was suspended with 1mL Trizol to extract nuclear RNA. Nuclear and cytoplasmic RNA were extracted with Trizol (TaKaRa, Kyoto, Japan) according to the standard protocol. The distributions of U1, GAPDH and circSnd1 were analyzed by RT-PCR.

**1.14 RNase R treatment**

Total RNA from C2C12 cells was extracted by Trizol (TaKaRa, Kyoto, Japan). 1000ng RNA was digested with with 3U RNase R (Epicentre, San Diego, CA, USA) at 37℃ for 0, 5, 10 and 15 min. RNase R was deactivated by heating at 85°C for 5 min, followed by the evaluation of circSnd1 expression using qRT-PCR.

**1.15 Fluorescence in situ hybridization** (**FISH)**

C2C12 cells were inoculated into a µ-Slide 8-well glass plate (density 5000/mL) and washed twice with 1×PBS 24 h later. The cells were treated with 4% PFA and fixed for 30 min at room temperature. 0.5% Triton X-100 and 2 mM ribonucleoside vanadyl complex (VRC) (NEB, Canada) were added and incubated on ice 10 min for permeabilization. After twice washing the cells in 2× saline sodium citrate (SSC) buffer, the cells were placed in a hybridization oven at 37°C overnight. The hybridization solution included 50ng/ml of probe. The next day, CY3-labeled streptavidin was added to the cells at room temperature and incubated for one hour following a wash with SSC buffer. The nuclei were stained by Hoechst staining solution (KeyGEN, Nanjing, China). The images were taken with a confocal microscope (Zeiss, Germany) under a 60× oil lens.

Following probes used as follows

Sense-probe-for-circSnd1: Biotin-5’- aaaCCTCTGTTGTATGTCGGGTCTCGTCTTGGC -3’;

Antisense-probe-for-circSnd11: Biotin-5’- aaaCGGTTCTGCTCTGGGCTGTATGTTGTCTCC -3’.

**1.16 Plasmid construction**

For the construction of overexpression plasmid, sequences of circSnd1 were cloned into the circular overexpression vector plasmid pK5ssAAV-ciR (Geneseed Biotech). CircSnd1 overexpressed plasmid sequence was synthesized by BGI (Shenzhen). For the construction of knockdown plasmids, the shRNA sequence was cloned into pENN.AAV.U6.ShRLuc.CMV.EGFP.SV40 plasmid.

The plasmid sequence was as follows:

circSnd1-OE-BamHI-Forward:

5’-CGCAATTGTAATACTTTCAGCCCAGAGCAGAACCG-3’

circSnd1-OE-BamHI-Reserves:

5’-CGGGATCCAGTTGTTCTTACCTGTATGTTGTCTCCCTCCA-3’

Sh-circSnd1-BamHI-Forward:

5’-GATCAACATACAGCCCAGAGCAGCTCGAGCTGCTCTGGGCTGTATGTTT

TTTTG-3’

Sh-circSnd1-EcoRI-Reserves:

5’-AATTCAAAAAAACATACAGCCCAGAGCAGCTCGAGCTGCTCTGGGCTGT

ATGTT-3’

EEF1A1-OE-AgeI-Forward:

5’-CCGACCGGTAGAACGGTTATAAGTGCGGCAG-3’

EEF1A1-OE-EcoRI-Reserves:

5’-CCGGAATTCTCATTTAGCCTTCTGAGCTTTCTG-3’

Sh-EEF1A1-AgeI-Forward-1:

5’-CCGGGCGTGGTATCACTATTGACATCTCGAGATGTCAATAGTGATACCAC

GCTTTTTG-3’

Sh-EEF1A1-EcoRI-Reserves-1:

5’-AATTCAAAAAGCGTGGTATCACTATTGACATCTCGAGATGTCAATAGTGA CCACGC-3’

Sh-EEF1A1-AgeI-Forward-2:

5’-CCGGGCTGGAGCCAAGTGCTAATATCTCGAGATATTAGCACTTGGCTCCA

GCTTTTTG-3’

Sh-EEF1A1-EcoRI-Reserves-2:

5’-AATTCAAAAAGCTGGAGCCAAGTGCTAATATCTCGAGATATTAGCACTTG GCTCCAGC-3’

**1.17** **AAV8 packing and treatment**

**Plasmid transfection:** 4 million HEK293T cells were seeded in 10 cm cell culture dishes . 10 μg pK5ssAAV-ciR-circSnd1 or pK5ssAAV-ciR-control (pENN-sh-circSnd1 or pENN-sh-control), 10μg pAAV2/8 (Addgene, #112864), 10μg pAdDeltaF6 (Addgene, #112867) as well as 90 μg PEI (1 mg/ml) were mixed in 1 mL of serum-free medium and added into the cell culture dish with 9ml culture medium. After 12 h post-transfection, the fresh medium was replaced. Subsequently, both the virus in the cells and the supernatant were collected 48 h after the initial transfection.

**Virus collection in culture medium:** 25 mL 40% PEG-8000 was added into 100 mL cell supernatant, stirred at 4℃ overnight, centrifuged at 2800g at 4℃ for 15 min. The virus precipitate was resuspended by adding 1 mL of lysis buffer (150 mM NaCl, 20 mM tris pH8.0).

**Virus collection in cells:** The cells were collected with a spatula and resuspended with 5mL of cell lysis buffer at -80°C. Freeze-thaw cycle was repeated three times in the dry ice and 37°C water bath. Next, the virus suspension in the culture medium were mixed with the thawed and freezed cell suspension. 1 M MgCl_2_ and Benzonase (Merck, Darmstadt, Germany) was added to the final concentration of 1 mM and 250U/mL, respectively. The virus mixture was subsequently incubated at 37℃ for a duration of 45 min. Centrifugation was used to extract the supernatant, using 4°C and 4000 rpm for 30 min.

**Iodixanol gradient density centrifugation to purify virus:** Iodixanol (3.5 mL 60%, 3.5 mL 40%, 4mL 25% and 4mL 17%) was slowly added to the centrifuge tube from the bottom to the top. The concentrated supernatant and cell lysate were slowly added to the top of the centrifuge tube, and the centrifuge tube was filled with cell lysis buffer. Centrifuge at 60000 rpm at 4℃ for 2 h. 70Ti rotor was used, with acceleration 6 and deceleration 9 (Beckman Coulter, Brea, CA, USA). 40% concentration layer of iodixanol was absorbed with a syringe, transferred to a concentration tube, and centrifuged with 10 mL PBS at 4000 rpm for 20 min at 4°C, repeated three times. Centrifuge and concentrate the virus to 500-1000 μl.

**AAV8 treatment: AAV8 virus were collected and treated mice by a single intramuscular injection at 1×10^11^ vg/mouse.**

**1.18 Determination of muscle fiber type**

**In muscle fibre-type determination, the detail method was described in our prior research[1]. Among them, the key points are as follows: primary antibodies used as followed: MHCI (1:50, BA-F8, DSHB), MHCIIa (1:50, SC-71, DSHB) and MHCIIb (1:50, BF-F3, DSHB). The corresponding secondary antibodies used as follows: Alexa Fluor 350 anti-mouse IgG2b (A-21140, Thermo Fisher), Alexa Fluor 488 anti-mouse IgG1 (A-21121, Thermo Fisher) and Alexa Fluor 555 anti-mouse IgM (A-21426, Thermo Fisher).**

**References**

1. Li Q, Yin X, Wan W, Zhou Y, Wang S, Yan Y, et al. EIF4A3 Promotes Muscle Atrophy and Aging by Inhibiting the FAK Pathway Through NEDD9 mRNA Destabilization. J Cachexia Sarcopenia Muscle. 2025;16:e70010.

**2. Supplementary Figures and Legends**

**
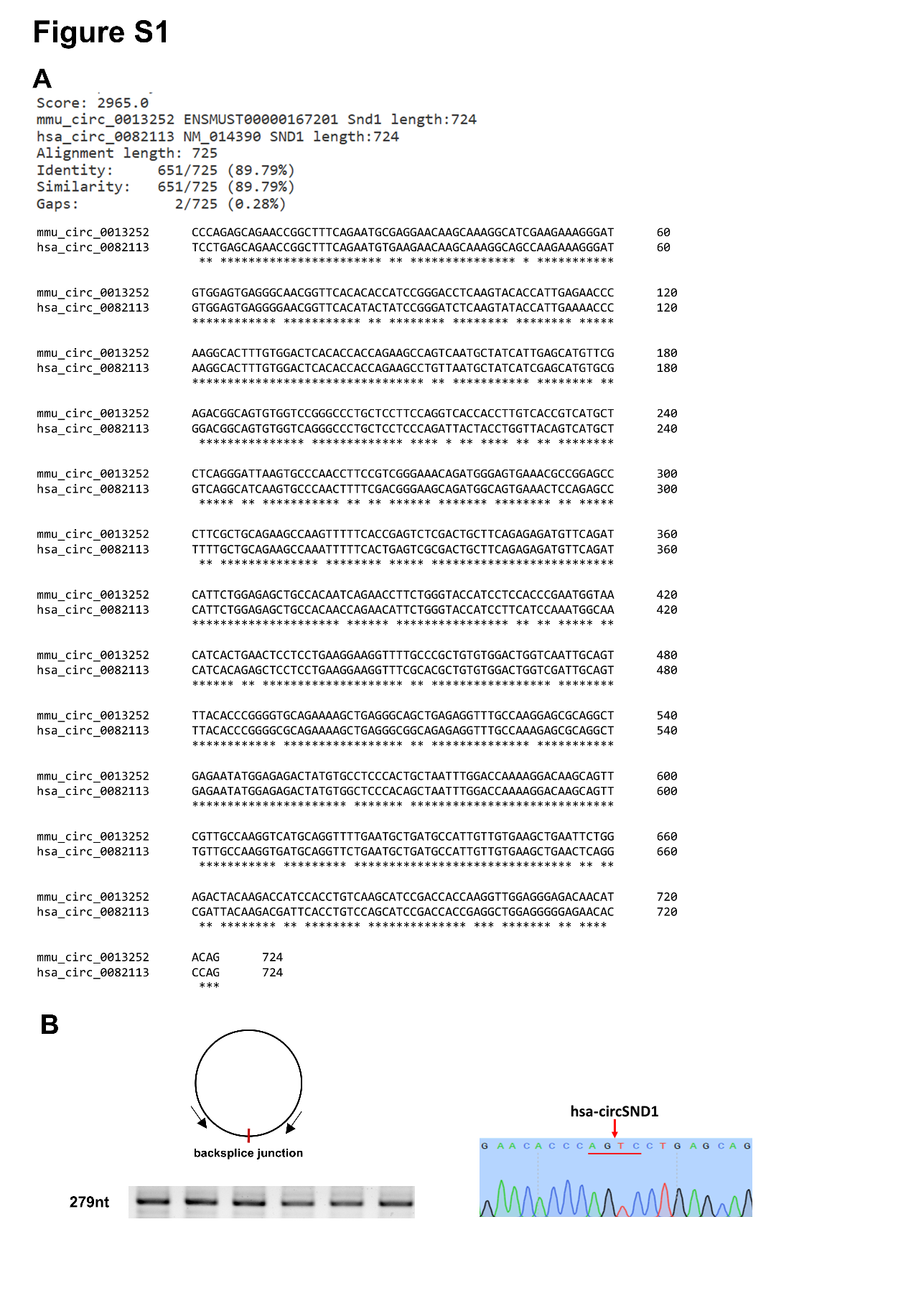
**

**Figure S1. circDdb1 (circBase ID: mmu_circ_0013252) is conserved between human (circBase ID: hsa_circ_0082113) and mouse species.**

**(A)Sequence alignment of *mmu*_circDdb1 and *hsa*_circSND1. (B)** **Analyzing the cirSND1 by performing gel electrophoresis and Sanger sequencing of the backsplice junction.**

**
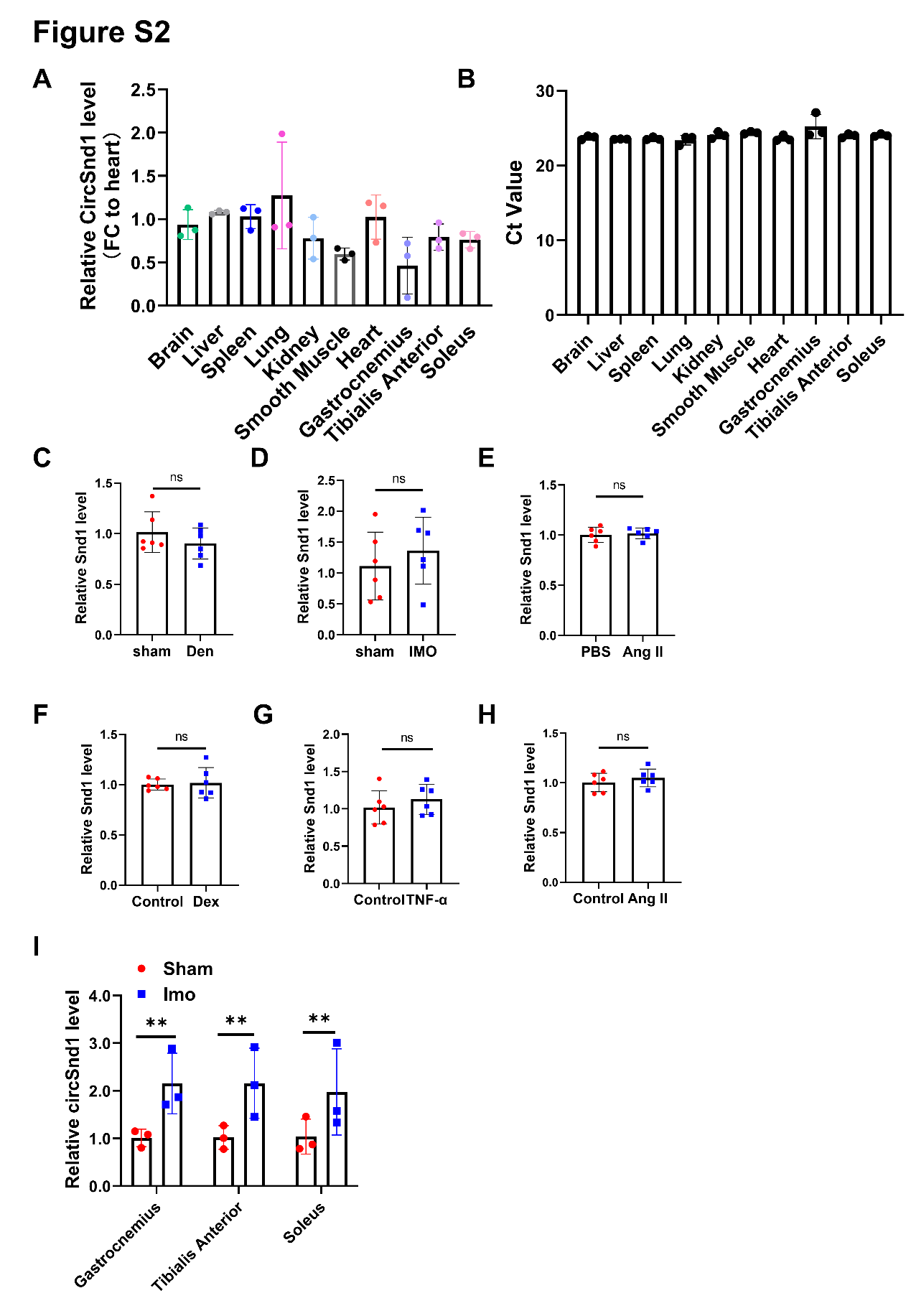
Figure S2 The circSnd1 and Snd1 content in muscle atrophy.**

**(A-B) Distribution of circSnd1 in different tissues and organs (n=3 per group).** (C-E) mRNA level of Snd1 in gastrocnemius muscle atrophy model including the treatment of denervation (Den), immobilization (Imo) as well as Angiotensin II (AngII) (n = 6 per group). (F-H) mRNA level of Snd1 in C2C12 myotubes with the treatment of Dexamethasone (Dex), tumor necrosis factor alpha (TNF-α) and AngII (n = 6 per group). **(I) RNA of circSnd1 in different types of skeletal muscle (Type I: soleus; Type II: gastrocnemius; Combination of TypeII and Type I: tibialis anterior) in muscle atrophy model induced by immobilization (n = 3 per group).** An unpaired, two-tailed Student’s t test was used for comparisons between two groups. **, p < 0.01. Data are represented as mean ± SD.

**
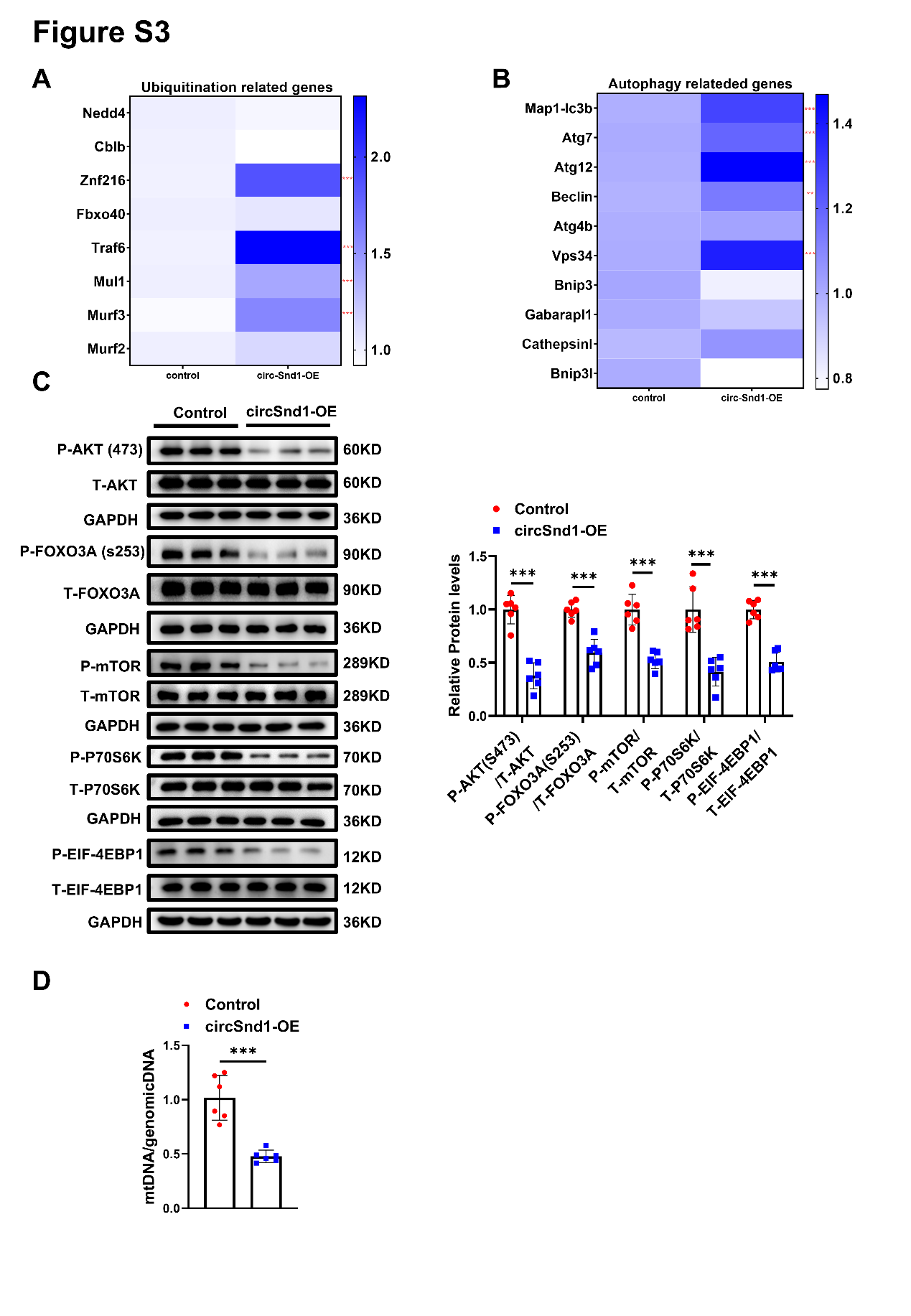
Figure S3** **circSnd1 promotes muscle atrophy *in*** ***vitro*.**

(A) mRNA level of UPS-related genes in circSnd1-OE (stimulated by circSnd1 overexpression plasmid) C2C12 myotubes compared to control group (n = 6 per group). (B) mRNA level of autophagy-related genes in circSnd1-OE C2C12 myotubes compared to control group (n = 6 per group). (C) Protein level of AKT, FOXO3A, mTOR, P70S6K and4EBP1 expression in circSnd1-OE C2C12 myotubes compared to control group (n = 6 per group). (D) The content of mitochondrial DNA (mtDNA) normalized by genomic DNA in circSnd1-OE C2C12 myotubes compared to control group (n = 6 per group). An unpaired, two-tailed Student’s t test was used for comparisons between two groups. **, p < 0.01; ***, p < 0.001. Data are represented as mean ± SD.

**
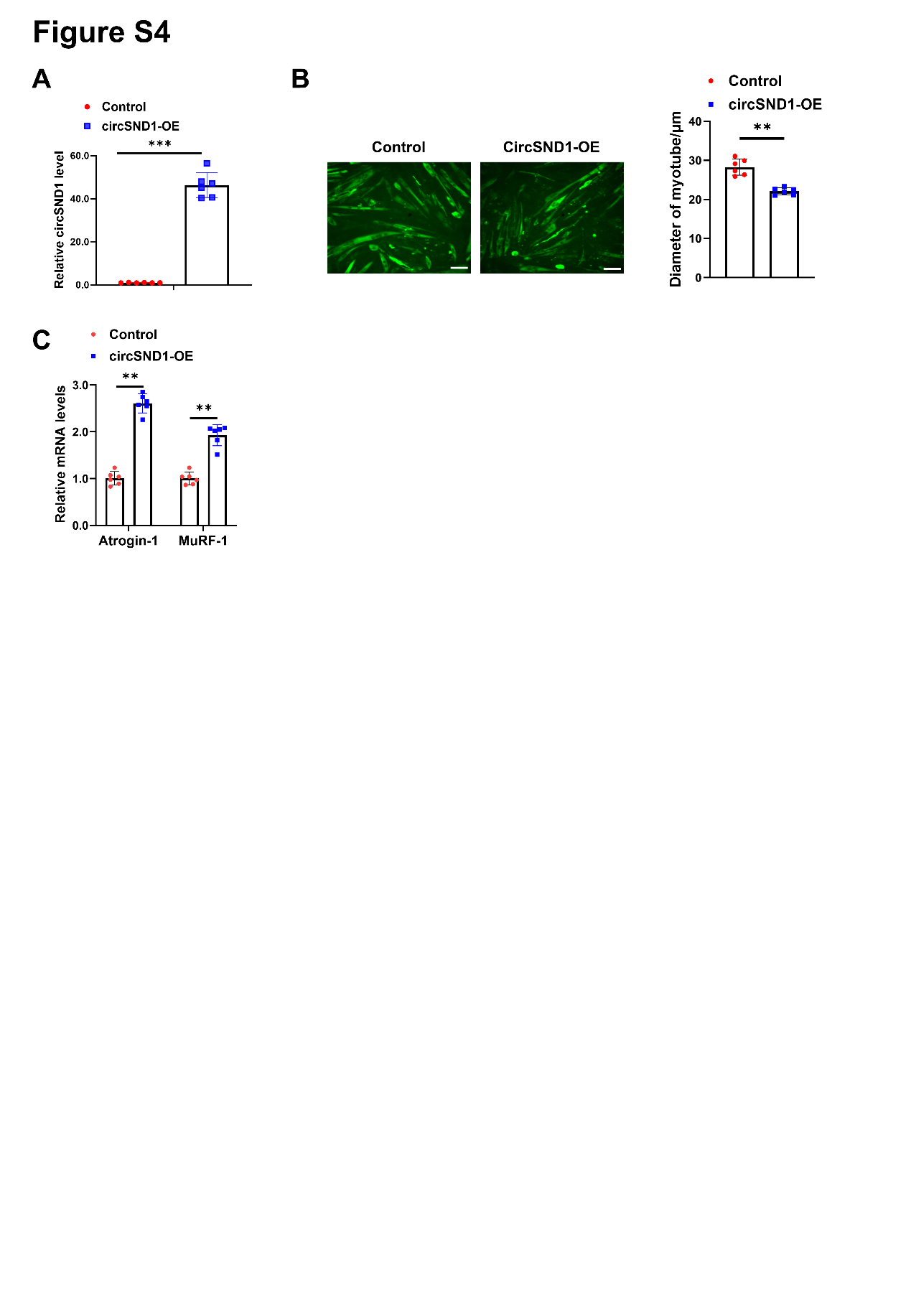
Figure S4 CircSND1 promotes muscle atrophy in human myotube *in vitro*.**

**(A)** **RNA levels of circSND1 expression in circSND1-OE plasmid treated human myotubes compared to control group (n = 6 per group). (B) Representative images and statistical analysis of human myotubes transfected with circSND1-OE and control plasmid (n = 6; scale bar: 50 μm). (C) Expression levels of MuRF-1 and Atrogin-1 mRNA levels in human myotube transfected with circSND1-OE and controls plasmid (n = 6). An unpaired, two-tailed Student’s t test was used for comparisons between two groups. **, p < 0.01; ***, p < 0.001. Data are represented as mean±SD.**

**
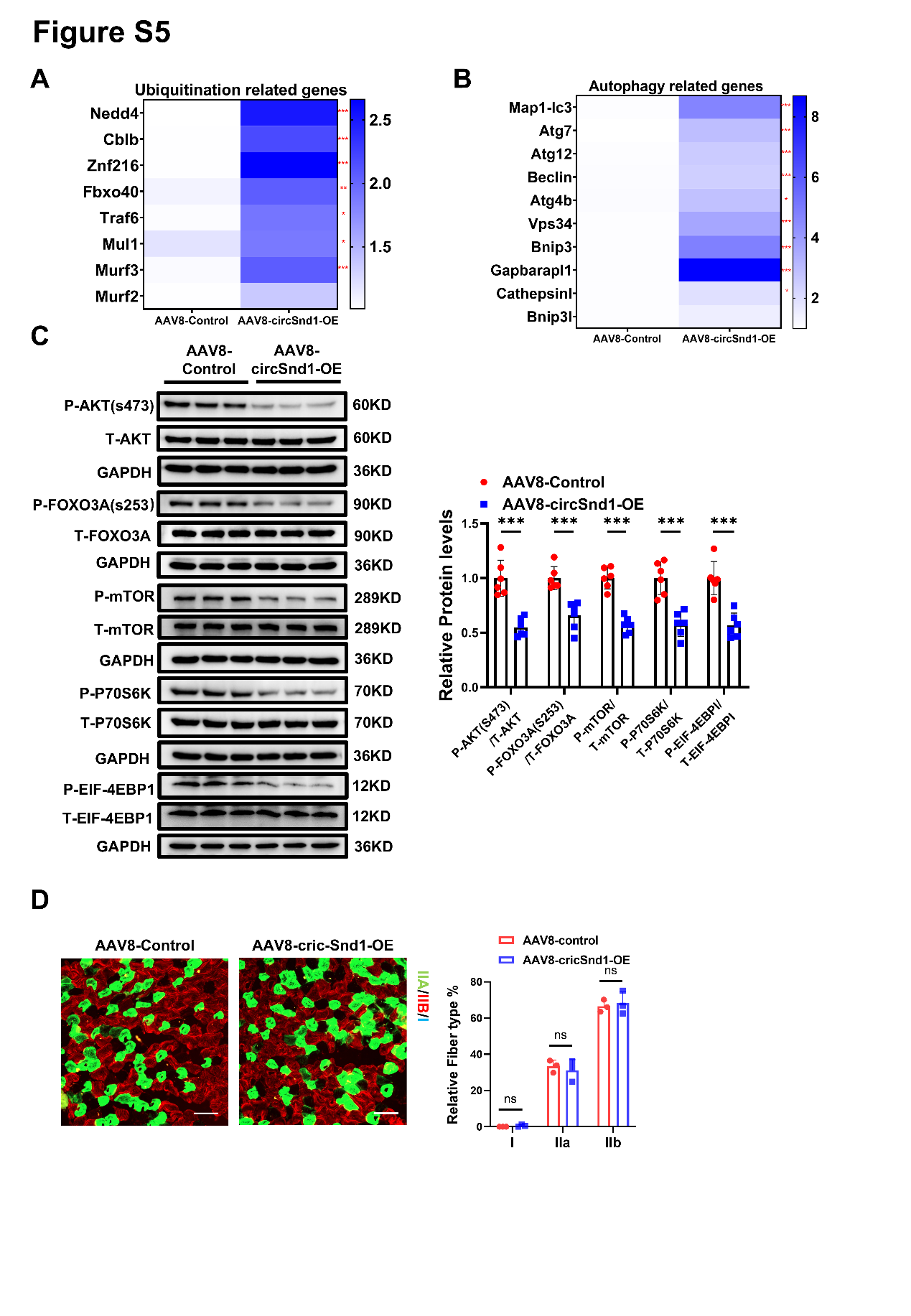
Figure S5** **circSnd1 promotes muscle atrophy *in vivo*.**

(A) mRNA level of UPS-related genes in mouse gastrocnemius muscle treated with circSnd1-OE (circSnd1 overexpression) AAV8 (AAV8- circSnd1-OE) compared to the control AAV8 (n = 10, 8). (B) mRNA level of autophagy-related genes in mouse gastrocnemius muscle treated with AAV8- circSnd1-OE compared to control AAV8(n = 10, 8). (C) Protein level of AKT, FOXO3A, mTOR, P70S6K and4EBP1 in mouse gastrocnemius muscle with AAV8-circSnd1-OE injection compared to AAV8-control (n = 6 per group). **(D) The types of gastrocnemius fibers in mice injected with AAV8-circSnd1-OE and AAV8-control were detected by immunofluorescence staining (n=3 per group; scale bar: 100 μm).** An unpaired, two-tailed Student’s t test was used for comparisons between two groups. *, p < 0.05; **, p < 0.01; ***, p <0.001. Data are represented as mean ± SD.

**
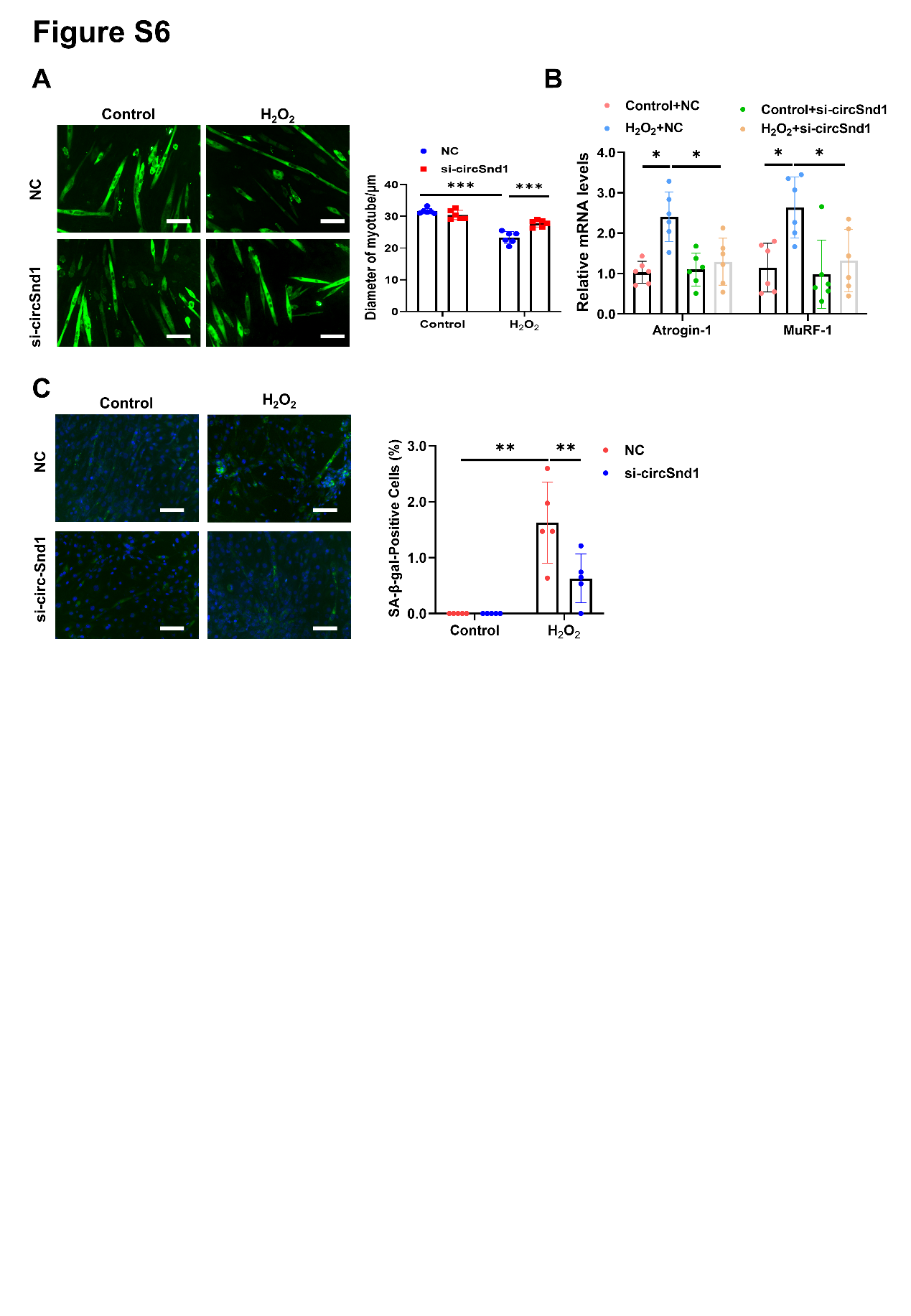
Figure S6 Inhibition of circSnd1 expression prevents H_2_O_2_-induced muscle atrophy and muscle aging.**

(A) Immunofluorescent staining and the diameter of C2C12 myotubes transfected with si-circSnd1 in H_2_O_2_-induced muscle-atrophy model. (n = 6 per group; scale bar: 50 μm); (B) mRNA levels of MuRF-1 and Atrogin-1 (n = 6 per group) in C2C12 myotubes transfected with si-circSnd1 H_2_O_2_-induced muscle-atrophy model. (C) SA-β-gal staining in C2C12 myotubes transfected with si-circSnd1in H_2_O_2_-induced muscle-atrophy model (n=5 per group; scale bar: 50 μm). Two-way ANOVA with Tukey test was performed. *, p < 0.05; **, p < 0.01; ***, p < 0.001. Data are represented as mean ± SD.

**
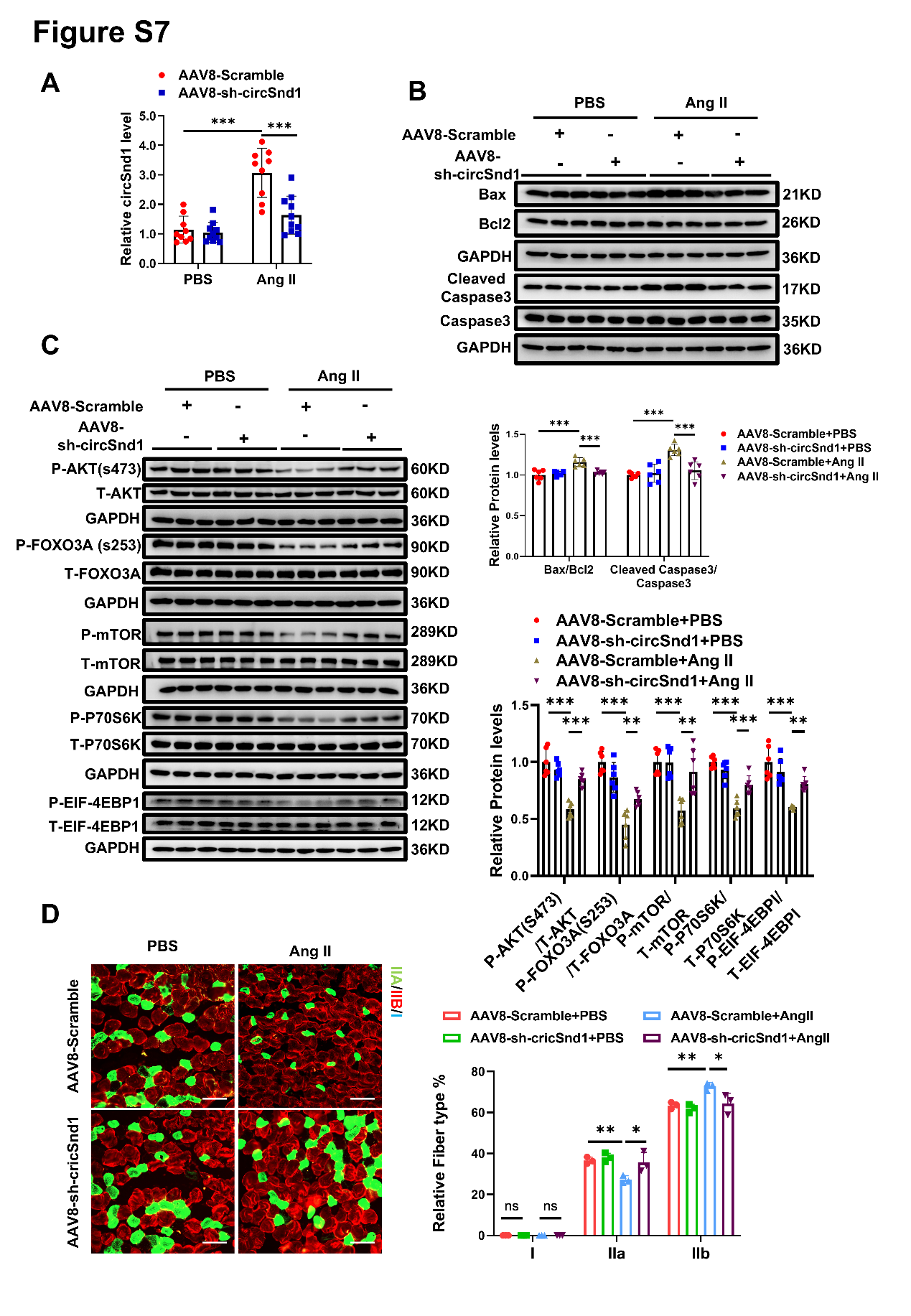
Figure S7** **Inhibition of circSnd1 prevents** **Angiotensin II (Ang II) induced muscle atrophy *in vivo*.**

(A) RNA level of circSnd1 expression in mouse gastrocnemius muscle after the injection of AAV8-sh-circSnd1 compared to AAV8-Scramble under the treatment of angiotensin II (AngII) (n =9, 10, 9,10). (B) Protein level of Bax, Bcl2 and Caspase3 expression in mice injected with AAV8-sh-circSnd1 compared to AAV8-Scramble under the treatment of Ang II (n = 6 per group). (C) Protein level of AKT, FOXO3A, mTOR, P70S6K and 4EBP1 expression in mice injected with AAV8-sh-circSnd1compared to AAV8-Scramble under the treatment of Ang II (n = 6 per group). **(D) The types of gastrocnemius fibers in muscle of mice injected with AAV8-sh-circSnd1 and AAV8-Scramble were detected by immunofluorescence staining (n=3; scale bar: 100 μm).** Two-way ANOVA with Tukey test was performed. ***, p < 0.05; **, p < 0.01;** ***, p < 0.001. Data are represented as mean ± SD.

**
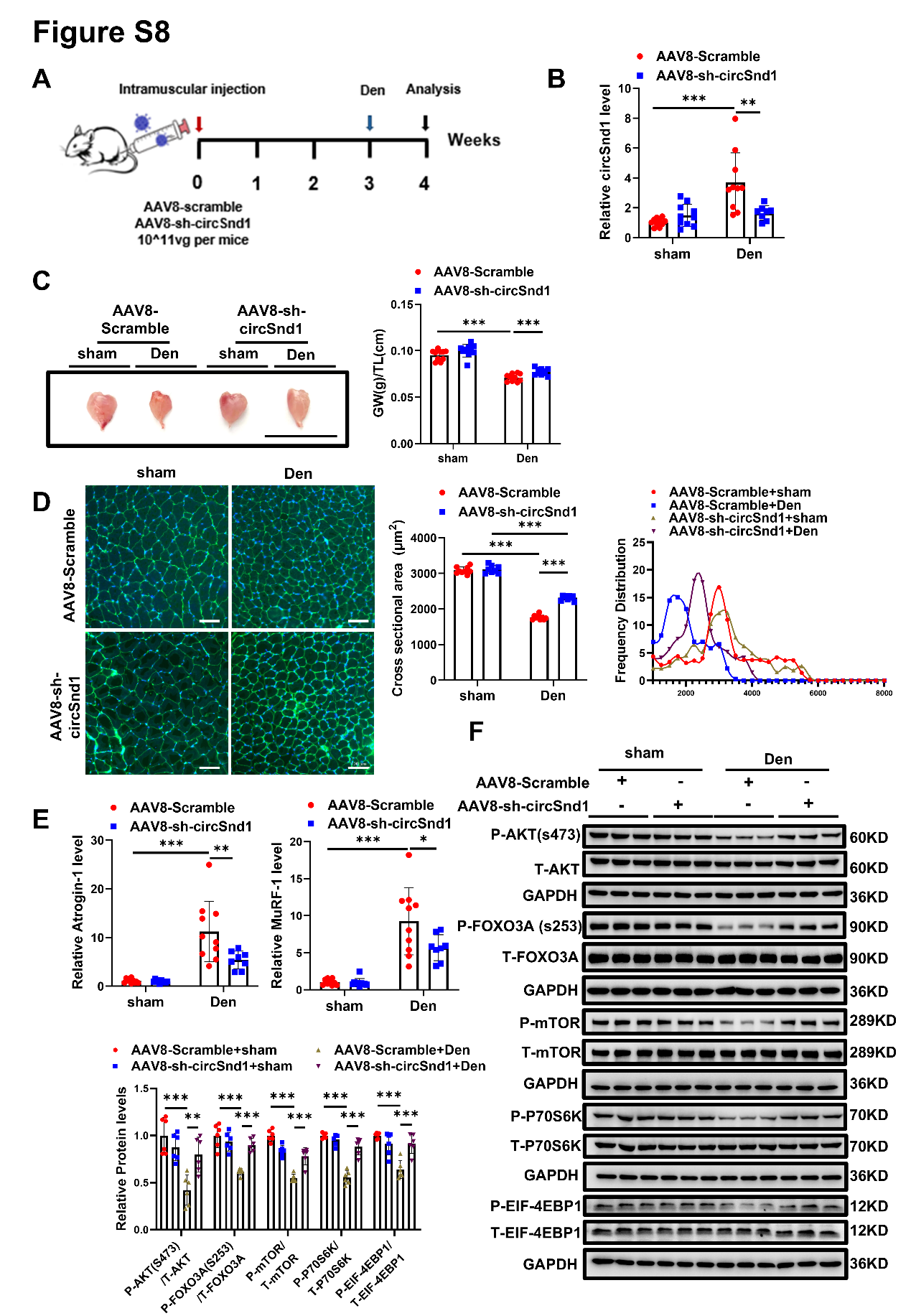
Figure S8 Inhibition of circSnd1 prevents denervation induced muscle atrophy *in vivo*.**

(A) Schematic diagram of experimental design. (B) RNA level of circSnd1 expression in mouse gastrocnemius muscle after the injection of AAV8-sh-circSnd1 compared to AAV8-Scrambleunder the treatment of denervation (Den) (n = 10, 10, 10, 8). (C) Gastrocnemius muscle morphology and **the ratio of gastrocnemius weight (GW) to tibia length (TL)** in mice with AAV8-sh-circSnd1 injection compared to AAV8-Scramble under the treatment of denervation (Den) (n = 10, 10, 10, 10; scale bar, 1 cm). (D) WGA staining for myofiber in mice with AAV8-sh-circSnd1 injection compared to AAV8-Scramble under the treatment of denervation (Den) (n = 9, 8, 9, 9; scale bar: 100 μm). (E) mRNA levels of MuRF-1and Atrogin-1 expression in mice with AAV8-sh-circSnd1 injection compared to AAV8-Scramble under the treatment of denervation (Den) (n = 10, 10, 10, 8). (F) Protein level of AKT, FOXO3A, mTOR, P70S6K and 4EBP1 expression in mice with AAV8-sh-circSnd1 injection compared to AAV8-Scramble under the treatment of denervation (Den) (n = 6 per group). Two-way ANOVA with Tukey test was performed (B-F). *, p < 0.05; **, p < 0.01; ***, p<0.001. Data were represented as mean ± SD.

**
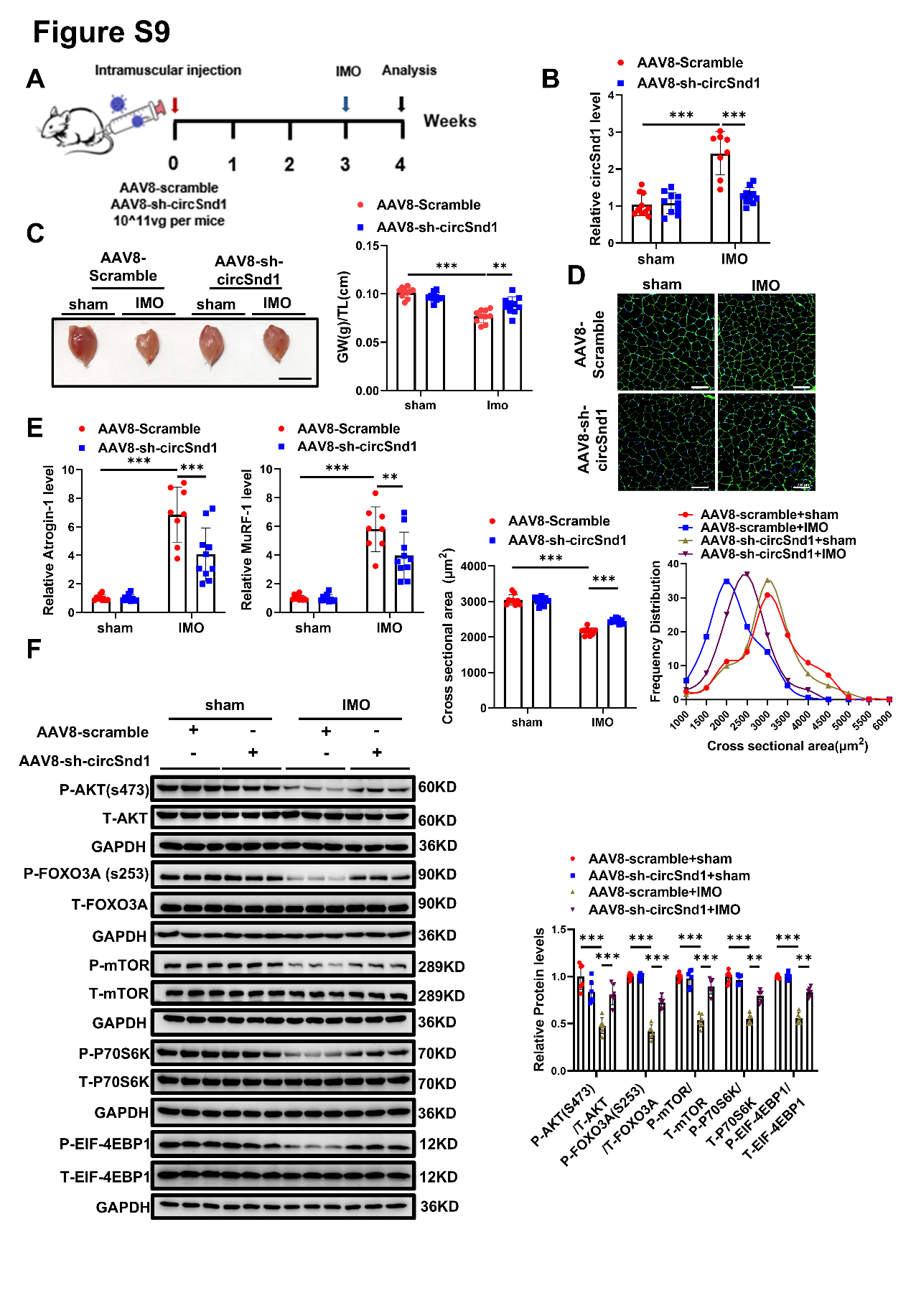
Figure S9** **Inhibition of circSnd1 prevents immobilization induced muscle atrophy *in vivo*.**

(A) Schematic diagram of experimental design. (B) RNA level of circSnd1 expression in mouse gastrocnemius muscle after the injection of AAV8-sh-circSnd1 compared to AAV8-Scramble under the treatment of immobilization (Imo) (n = 10, 10, 8, 10). (C) The mouse gastrocnemius muscle morphology and **the ratio of gastrocnemius weight (GW)to tibia length (TL)** of mice after the injection of AAV8-sh-circSnd1 compared to AAV8-Scramble under the treatment of immobilization (Imo) (n = 10 per group; scale bar: 1 cm). (D) WGA staining for mouse myofiber with the injection of AAV8-sh-circSnd1 compared to AAV8-Scramble under the treatment of immobilization (Imo) (n = 9, 10, 9, 9; scale bar: 100 μm). (E) mRNA level of MuRF-1 and Atrogin-1 expression in mouse gastrocnemius muscle after the injection of AAV8-sh-circSnd1 compared to AAV8-Scramble under the treatment of immobilization (Imo) (n = 10, 10, 8, 10). (F) Protein level of AKT, FOXO3A, mTOR, P70S6K and 4EBP1 expression in mice injected with AAV8-sh-circSnd1compared to AAV8-Scramble in muscle atrophy induced by immobilization (Imo) (n = 6 per group). Two-way ANOVA with Tukey test was performed (B-F). **, p < 0.01; ***, p<0.001. Data were represented as mean ± SD.

**
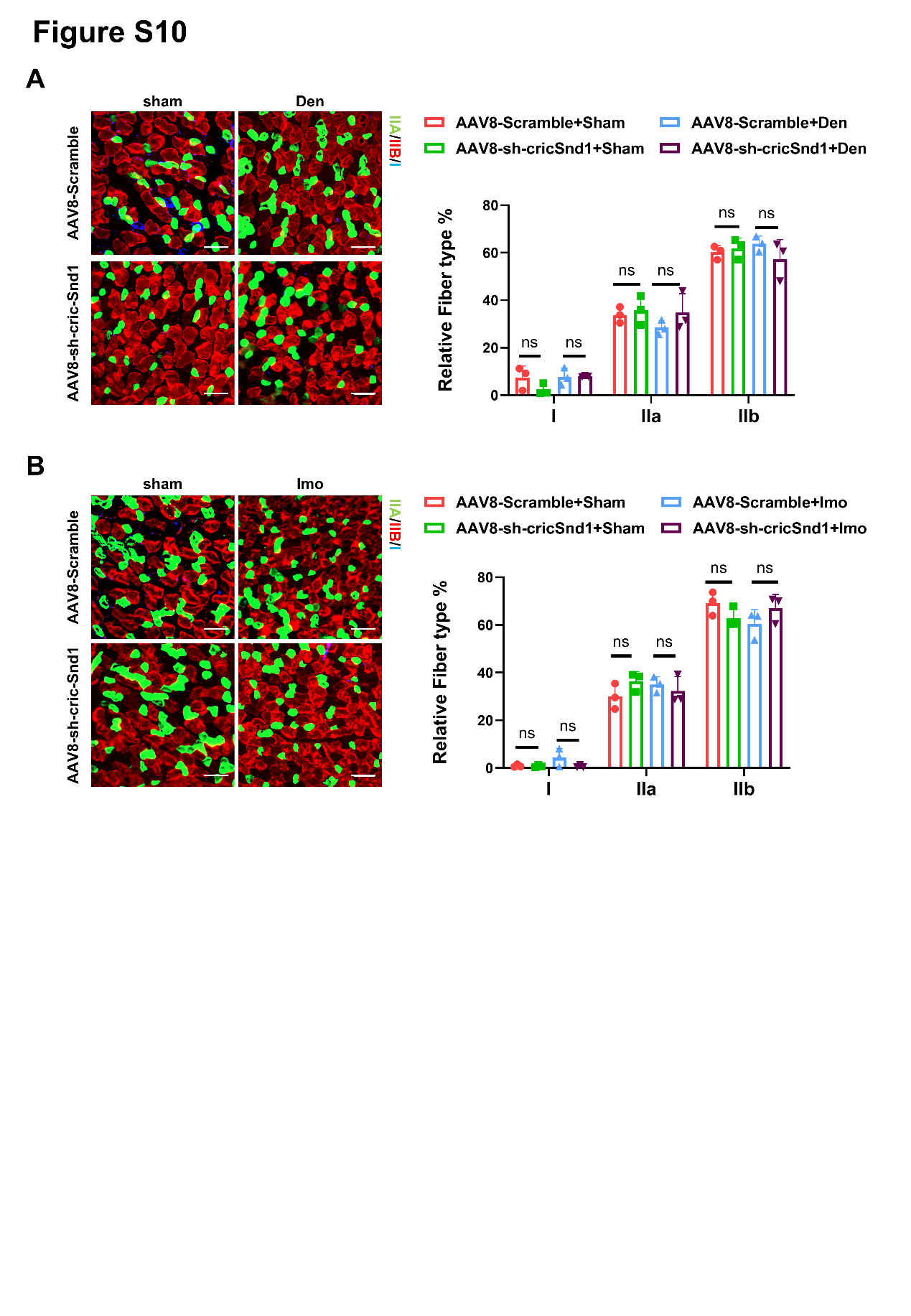
Figure S10 Inhibition of circSnd1 doesn’t change the muscle fiber composition in denervation and immobilization induced muscle atrophy *in vivo*.**

**The types of gastrocnemius fibers in muscle of mice injected with AAV8-sh-circSnd1 and AAV8-Scramble in denervation (A) and immobilization (B) induced muscle atrophy, which were detected by immunofluorescence staining (n=3; scale bar: 100 μm).** Two-way ANOVA with Tukey test was performed. Data are represented as mean ± SD.

**
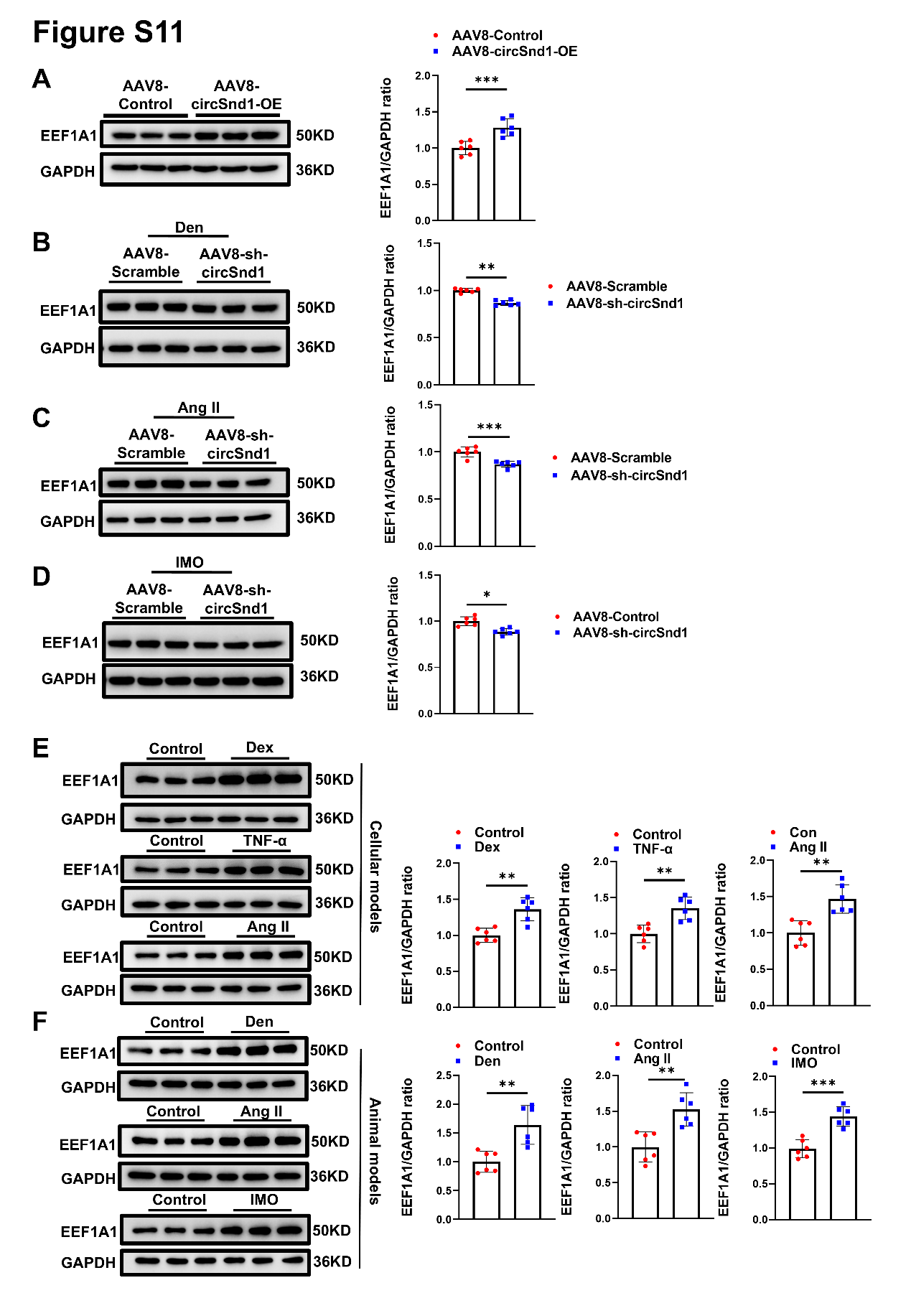
 Figure S11** **EEF1A1 is upregulated in muscle atrophy.**

(A) Protein level of EEF1A1 expression in circSnd1-OE (stimulated by circSnd1 overexpression plasmid) C2C12 myotubes compared to control group (n = 6 per group). (B) Protein level of EEF1A1 expression in mouse gastrocnemius muscle injected with AAV8-sh-circSnd1 compared to AAV8-Scramble in Den-induced muscle atrophy (n = 6 per group). (C) Protein level of EEF1A1 expression in mouse gastrocnemius muscle injected with AAV8-sh-circSnd1 compared to AAV8-Scramble in AngII-induced muscle atrophy (n = 6 per group). (D) Protein level of EEF1A1 expression in mouse gastrocnemius muscle injected with AAV8-sh-circSnd1 compared to AAV8-Scramble in IMO-induced muscle atrophy (n = 6 per group). (E) Protein level of EEF1A1 expression in **C2C12 myotube** treated with Dex-, TNFα- as well as AngII (n = 6 per group). (F) Protein level of EEF1A1 expression in mouse gastrocnemius muscle samples treated with Den-, AngII- as well as IMO (n = 6 per group). An unpaired, two-tailed Student’s t test was used in (A, E and F), Two-way ANOVA with Tukey test was performed in (B, C and D). *, p < 0.05; **, p < 0.01; ***, p < 0.001. Data are represented as mean ± SD.

**
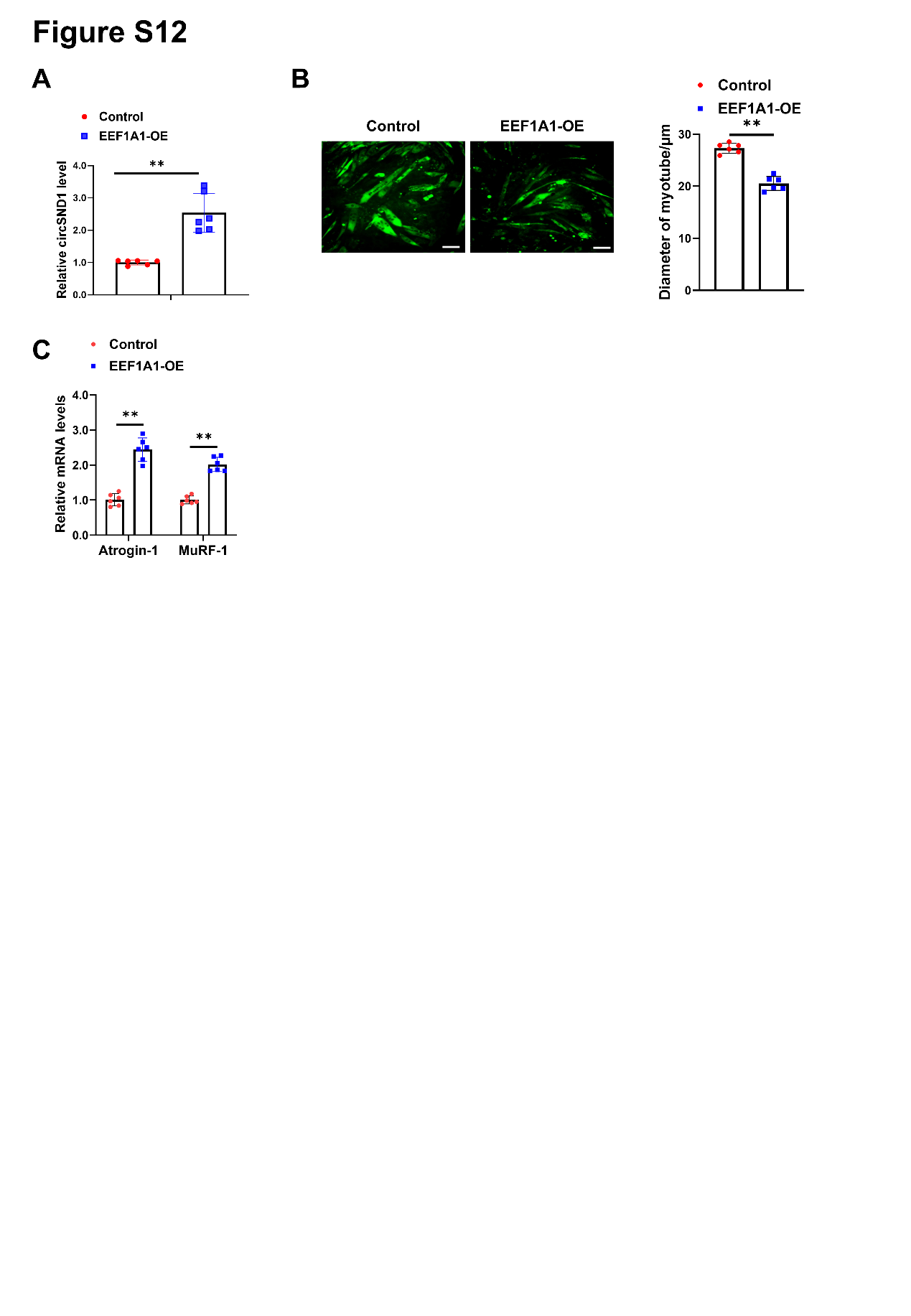
Figure S12 EEF1A1 promotes muscle atrophy *in vitro*.**

**(A)** **mRNA levels of EEF1A1 expression in EEF1A1-OE lentivirus treated C2C12 myotubes compared to control group (n = 6 per group). (B) Representative images and statistical analysis of C2C12 myotubes transfected with EEF1A1-OE and control lentivirus (n = 6; scale bar: 50 μm). (C) Expression levels of MuRF-1 and Atrogin-1 mRNA levels in C2C12 myotube transfected with EEF1A1-OE and controls lentivirus (n = 6). An unpaired, two-tailed Student’s t test was used for comparisons between two groups. **, p < 0.01. Data are represented as mean±SD.**

**
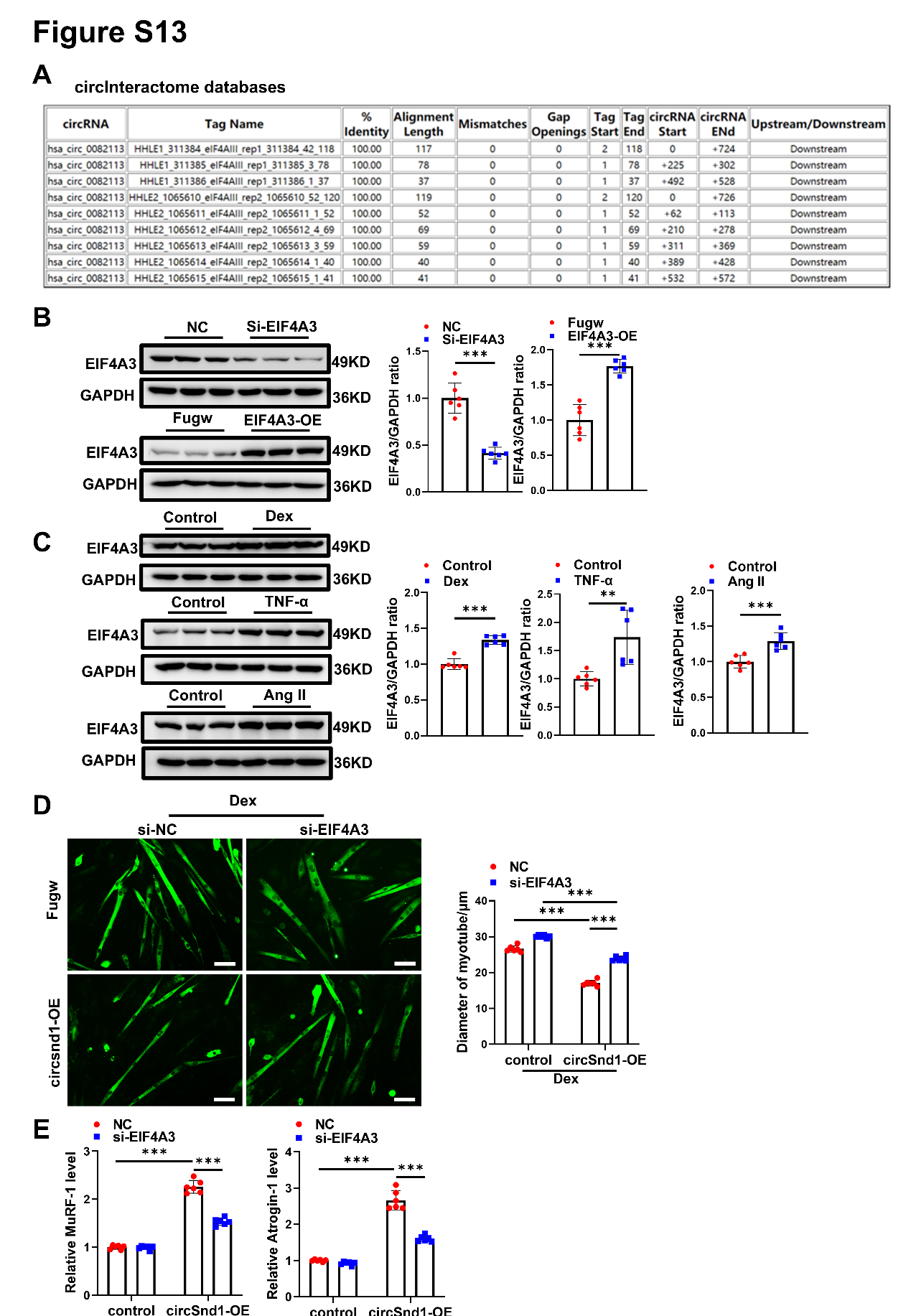
Figure S13. EIF4A3 induces circSnd1 expression in muscle atrophy.**

**(A)** The RBP of flanking sequences of circSnd1 were predicted using CircInteractome databases. **(B)** Protein level of EEF1A1 expression in C2C12 myotube cells treated with EIF4A3 overexpression plasmid and si-EIF4A3 compared to control (n=6). **(C)** Protein level of EIF4A3 expression in mouse gastrocnemius muscle samples from Dex-, TNFα-, AngII-induced muscle atrophy model (n = 6 per group). **(D)** Immunofluorescent staining and quantification of the diameter of C2C12 myotubes transfected with circSnd1-OE plasmid, si-EIF4A3 under dexamethasone (Dex) treatment. (n = 6 per group; scale bar: 50 μm). **(E)** mRNA level of MuRF-1 and Atrogin-1 expression (n = 6 per group) in C2C12 myotubes transfected with circSnd1-OE plasmid, si-EIF4A3 under dexamethasone (Dex) treatment (n = 6 per group). An unpaired, two-tailed Student’s t test was used in (B and C), Two-way ANOVA with Tukey test was performed (D and E). **, p < 0.01; ***, p < 0.001. Data are represented as mean ± SD.

**
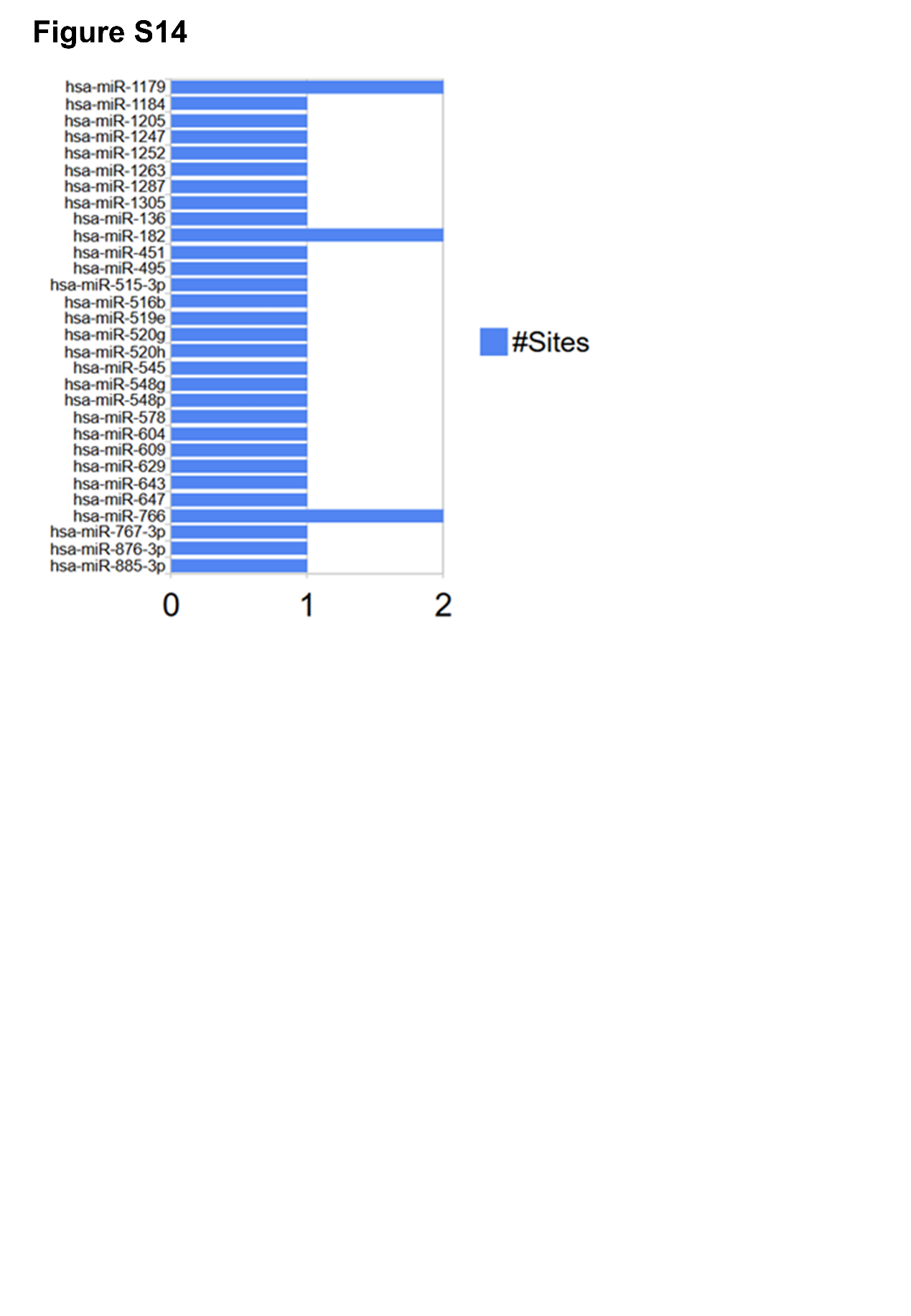
Figure S14 The number of binding sites for miRNAs in circSnd1.**

**The binding between circSnd1 and miRNAs was predicted using CircInteractome databases.**

**
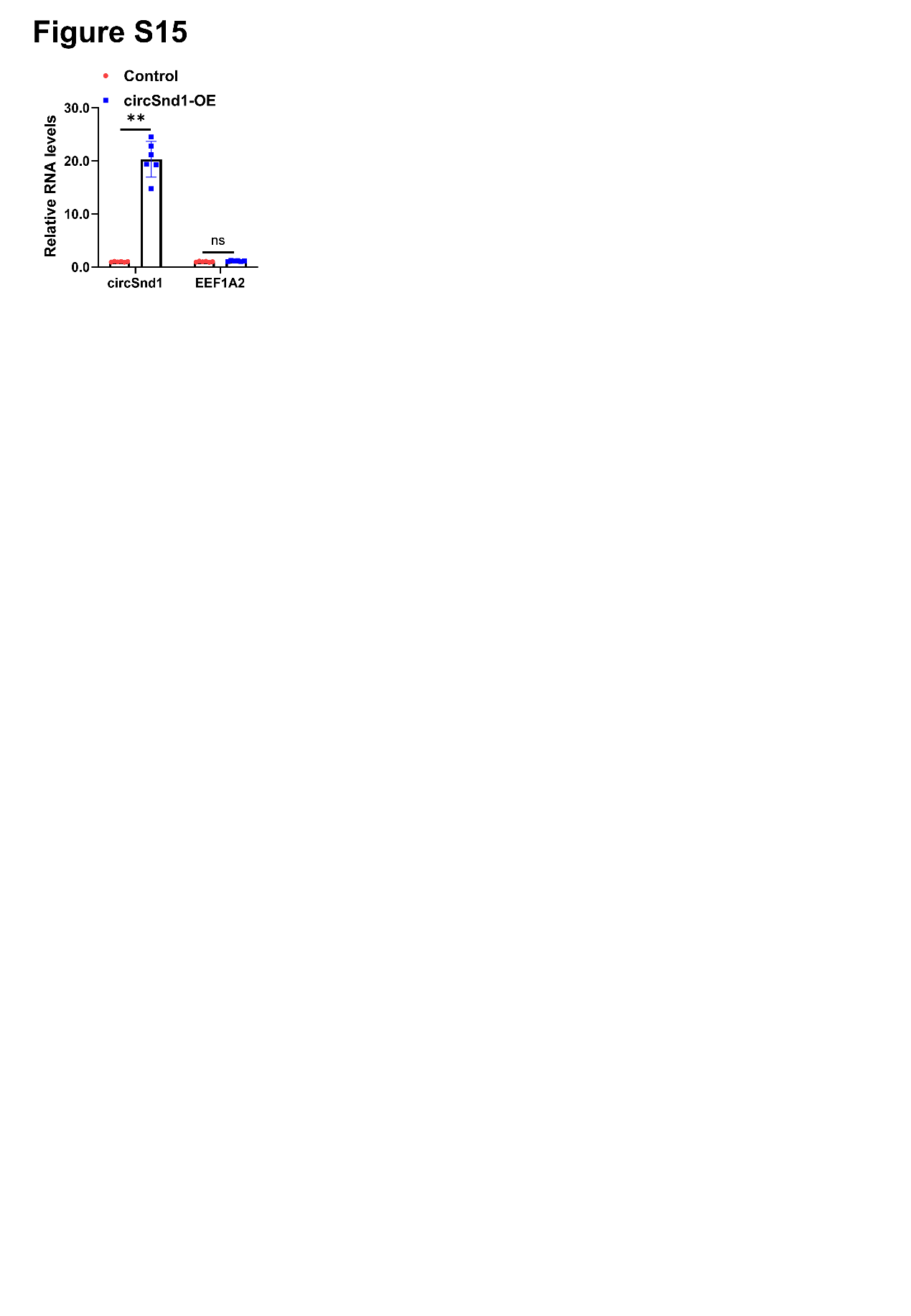
Figure S15 circSnd1 doesn’t change the EEF1A2 mRNA expression.**

**Expression levels of circSnd1 and EEF1A2 RNA levels in C2C12 myotube transfected with circSnd1-OE and controls plasmid (n = 6). An unpaired, two-tailed Student’s t test was used for comparisons between two groups. **, p < 0.01; Data are represented as mean±SD.**

**Supplementary Tables:**

**Table S****1 Primer for RT-qPCR**

| **Primer for RT-qPCR** | **Sequences (5’-3’)** |
| --- | --- |
| mmu-circSnd1-F | ATCCACCTGTCAAGCAT |
| mmu-circSnd1-R | GACGGAAGGTTGGGCA |
| mmu-Snd1-F | TGTTGTCGACTCCAGACCTG |
| mmu-Snd1-R | ACACTTGGGAAGAAGGGTGG |
| hsa-circSND1-F | AAAGAGCGCAGGCTGAGAATATGGA |
| hsa-circSND1-F | CAAAGTGCCTTGGGTTTTCAATGGT |
| mmu-EIF4A3-F | CACCAACGTCGGCGAGGA |
| mmu- EIF4A3-R | GATGGCCACACCTTTTCGTC |
| mmu-Map1-lc3-F | CACTGCTCTGTCTTGTGTAGGTTG |
| mmu-Map1-lc3-R | TCGTTGTGCCTTTATTAGTGCATC |
| mmu-Atg7-F | GTTCGCCCCCTTTAATAGTGC |
| mmu-Atg7-R | TGAACTCCAACGTCAAGCGG |
| mmu-Atg12-F | TCCGTGCCATCACATACACA |
| mmu-Atg12-R | TAAGACTGCTGTGGGGCTGA |
| mmu-Beclin-F | TGAATGAGGATGACAGTGAGCA |
| mmu-Beclin-R | CACCTGGTTCTCCACACTCTTG |
| mmu-Atg4b-F | ATTGCTGTGGGGTTTTTCTG |
| mmu-Atg4b-R | AACCCCAGGATTTTCAGAGG |
| mmu-Vps34-F | TGTCAGATGAGGAGGCTGTG |
| mmu-Vps34-R | CCAGGCACGACGTAACTTCT |
| mmu-Bnip3-F | TTCCACTAGCACCTTCTGATGA |
| mmu-Bnip3-R | GAACACCGCATTTACAGAACAA |
| mmu-Gabarapl1-F | CATCGTGGAGAAGGCTCCTA |
| mmu-Gabarapl1-R | ATACAGCTGGCCCATGGTAG |
| mmu-Cathepsinl-F | GTGGACTGTTCTCACGCTCAAG |
| mmu-Cathepsinl-R | TCCGTCCTTCGCTTCATAGG |
| mmu-Bnip3l-F | TTGGGGCATTTTACTAACCTTG |
| mmu-Bnip3l-R | TGCAGGTGACTGGTGGTACTAA |
| mmu-Nedd4-F | TCGGAGGACGAGGTATGGG |
| mmu-Nedd4-R | GGTACGGATCAGCAGTGAACA |
| mmu-Cblb-F | GGTCGCATTTTGGGGATTATTGA |
| mmu-Cblb-R | TTTGGCACAGTCTTACCACTTT |
| mmu-Znf216-F | CCCATGCTGTGTAGTACAGGA |
| mmu-Znf216-R | GCTCATTCTGCCACTATTCTGC |
| mmu-Fbxo40-F | CGTCTCCTGCCTGGTGATAAG |
| mmu-Fbxo40-R | GTATGCTCTGACTCTTTGCACAT |
| mmu-Traf6-F | AAAGCGAGAGATTCTTTCCCTG |
| mmu-Traf6-R | ACTGGGGACAATTCACTAGAGC |
| mmu-Mul1-F | CTGGGCACCAGTTCGATGG |
| mmu-Mul1-R | GACAGCATAAGGCACACACTT |
| mmu-Murf3-F | GGAGAAGCAGCTCATTTGCC |
| mmu-Murf3-R | CCTCCTGAAGACACCGTTGTG |
| mmu-Murf2-F | AAAGCAACTGATCTGTCCCATC |
| mmu-Murf2-R | TGTGGGTAAGTACGGGTTAGAG |
| mmu-18s-F | TCAAGAACGAAAGTCGGAGG |
| mmu-18s-R | GGACATCTAAGGGCATCAC |
| mmu-Atrogin-1-F | CAGCTTCGTGAGCGACCTC |
| mmu-Atrogin-1-R | GGCAGTCGAGAAGTCCAGTC |
| mmu-Murf-1-F | GTGTGAGGTGCCTACTTGCTC |
| mmu-Murf-1-R | GCTCAGTCTTCTGTCCTTGGA |
| mmu-Eef1a2-F | ATTGTGGGTGTCAACAAGATGG |
| mmu-Eef1a2-R | CATGGCATATTAGGTGAAGGCTC |

**Supplementary References:**

S1. Legnini I, Di Timoteo G, Rossi F, Morlando M, Briganti F, Sthandier O, et al. Circ-ZNF609 Is a Circular RNA that Can Be Translated and Functions in Myogenesis. Mol Cell. 2017;66:22-37 e9.

S2. Yan J, Yang Y, Fan X, Liang G, Wang Z, Li J, et al. circRNAome profiling reveals circFgfr2 regulates myogenesis and muscle regeneration via a feedback loop. J Cachexia Sarcopenia Muscle. 2022;13:696-712.

S3. Li M, Zhang N, Li J, Ji M, Zhao T, An J, et al. CircRNA Profiling of Skeletal Muscle in Two Pig Breeds Reveals CircIGF1R Regulates Myoblast Differentiation via miR-16. Int J Mol Sci. 2023;24:

S4. Shen X, Cui C, Tang S, Han S, Zhang Y, Xia L, et al. MyoG-enhanced circGPD2 regulates chicken skeletal muscle development by targeting miR-203a. Int J Biol Macromol. 2022;222:2212-24.

S5. Ashwal-Fluss R, Meyer M, Pamudurti NR, Ivanov A, Bartok O, Hanan M, et al. circRNA biogenesis competes with pre-mRNA splicing. Mol Cell. 2014;56:55-66.

S6. Zhao Q, Liu J, Deng H, Ma R, Liao JY, Liang H, et al. Targeting Mitochondria-Located circRNA SCAR Alleviates NASH via Reducing mROS Output. Cell. 2020;183:76-93 e22.

S7. Du WW, Yang W, Liu E, Yang Z, Dhaliwal P, Yang BB. Foxo3 circular RNA retards cell cycle progression via forming ternary complexes with p21 and CDK2. Nucleic Acids Res. 2016;44:2846-58.

S8. Huang S, Li X, Zheng H, Si X, Li B, Wei G, et al. Loss of Super-Enhancer-Regulated circRNA Nfix Induces Cardiac Regeneration After Myocardial Infarction in Adult Mice. Circulation. 2019;139:2857-76.

S9. Fei T, Chen Y, Xiao T, Li W, Cato L, Zhang P, et al. Genome-wide CRISPR screen identifies HNRNPL as a prostate cancer dependency regulating RNA splicing. Proc Natl Acad Sci U S A. 2017;114:E5207-E15.

S10. Errichelli L, Dini Modigliani S, Laneve P, Colantoni A, Legnini I, Capauto D, et al. FUS affects circular RNA expression in murine embryonic stem cell-derived motor neurons. Nat Commun. 2017;8:14741.

S11. Omata Y, Okawa M, Haraguchi M, Tsuruta A, Matsunaga N, Koyanagi S, Ohdo S. RNA editing enzyme ADAR1 controls miR-381-3p-mediated expression of multidrug resistance protein MRP4 via regulation of circRNA in human renal cells. J Biol Chem. 2022;298:102184.

S12. Conn SJ, Pillman KA, Toubia J, Conn VM, Salmanidis M, Phillips CA, et al. The RNA binding protein quaking regulates formation of circRNAs. Cell. 2015;160:1125-34.

S13. Li J, Chan MC, Yu Y, Bei Y, Chen P, Zhou Q, et al. miR-29b contributes to multiple types of muscle atrophy. Nature communications. 2017;8:15201.

S14. Li J, Wang L, Hua X, Tang H, Chen R, Yang T, et al. CRISPR/Cas9-Mediated miR-29b Editing as a Treatment of Different Types of Muscle Atrophy in Mice. Molecular therapy : the journal of the American Society of Gene Therapy. 2020;28:1359-72.

S15. Li J, Yang T, Tang H, Sha Z, Chen R, Chen L, et al. Inhibition of lncRNA MAAT Controls Multiple Types of Muscle Atrophy by cis- and trans-Regulatory Actions. Mol Ther. 2021;29:1102-19.

S16. Chen R, Yuan W, Zheng Y, Zhu X, Jin B, Yang T, et al. Delivery of engineered extracellular vesicles with miR-29b editing system for muscle atrophy therapy. J Nanobiotechnology. 2022;20:304.

S17. Liu Q, Yuan W, Yan Y, Jin B, You M, Liu T, et al. Identification of a novel small-molecule inhibitor of miR-29b attenuates muscle atrophy. Mol Ther Nucleic Acids. 2023;31:527-40.

S18. Liu Q, Chen L, Liang X, Cao Y, Zhu X, Wang S, et al. Exercise attenuates angiotensinⅡ-induced muscle atrophy by targeting PPARgamma/miR-29b. J Sport Health Sci. 2022;11:696-707.

S19. Zheng X, Huang M, Xing L, Yang R, Wang X, Jiang R, et al. The circRNA circSEPT9 mediated by E2F1 and EIF4A3 facilitates the carcinogenesis and development of triple-negative breast cancer. Mol Cancer. 2020;19:73.

S20. Brar HS, Greenspoon JS, Platt LD, Paul RH. Acute puerperal uterine inversion. New approaches to management. J Reprod Med. 1989;34:173-7.

S21. Feng ZH, Zheng L, Yao T, Tao SY, Wei XA, Zheng ZY, et al. EIF4A3-induced circular RNA PRKAR1B promotes osteosarcoma progression by miR-361-3p-mediated induction of FZD4 expression. Cell Death Dis. 2021;12:1025.

S22. Jiang X, Guo S, Wang S, Zhang Y, Chen H, Wang Y, et al. EIF4A3-Induced circARHGAP29 Promotes Aerobic Glycolysis in Docetaxel-Resistant Prostate Cancer through IGF2BP2/c-Myc/LDHA Signaling. Cancer Res. 2022;82:831-45.

S23. Scaggiante B, Dapas B, Bonin S, Grassi M, Zennaro C, Farra R, et al. Dissecting the expression of EEF1A1/2 genes in human prostate cancer cells: the potential of EEF1A2 as a hallmark for prostate transformation and progression. Br J Cancer. 2012;106:166-73.

S24. Leclercq TM, Moretti PA, Pitson SM. Guanine nucleotides regulate sphingosine kinase 1 activation by eukaryotic elongation factor 1A and provide a mechanism for eEF1A-associated oncogenesis. Oncogene. 2011;30:372-8.

S25. Lee S, Wolfraim LA, Wang E. Differential expression of S1 and elongation factor-1 alpha during rat development. J Biol Chem. 1993;268:24453-9.
